# An integrative “omics” approach identifies new candidate genes to impact aroma volatiles in peach fruit

**DOI:** 10.1186/1471-2164-14-343

**Published:** 2013-05-23

**Authors:** Gerardo Sánchez, Mónica Venegas-Calerón, Joaquín J Salas, Antonio Monforte, María L Badenes, Antonio Granell

**Affiliations:** 1 Instituto de Biología Molecular y Celular de Plantas (IBMCP), Ingeniero Fausto Elio s/n, Valencia 46022, Spain; 2 Instituto Nacional de Tecnología Agropecuaria (INTA), Ruta N°9 Km 170, San Pedro 2930, Argentine; 3 Instituto de la Grasa (IG-CSIC), Av. Padre García Tejero, 4, Sevilla 41012, Spain; 4 Instituto Valenciano de Investigaciones Agrarias (IVIA), Carretera Moncada-Náquera, Km 4,5, Valencia, Náquera 46113, Spain

## Abstract

**Background:**

Ever since the recent completion of the peach genome, the focus of genetic research in this area has turned to the identification of genes related to important traits, such as fruit aroma volatiles. Of the over 100 volatile compounds described in peach, lactones most likely have the strongest effect on fruit aroma, while esters, terpenoids, and aldehydes have minor, yet significant effects. The identification of key genes underlying the production of aroma compounds is of interest for any fruit-quality improvement strategy.

**Results:**

Volatile (52 compounds) and gene expression (4348 genes) levels were profiled in peach fruit from a maturity time-course series belonging to two peach genotypes that showed considerable differences in maturation characteristics and postharvest ripening. This data set was analyzed by complementary correlation-based approaches to discover the genes related to the main aroma-contributing compounds: lactones, esters, and phenolic volatiles, among others. As a case study, one of the candidate genes was cloned and expressed in yeast to show specificity as an ω-6 Oleate desaturase, which may be involved in the production of a precursor of lactones/esters.

**Conclusions:**

Our approach revealed a set of genes (an alcohol acyl transferase, fatty acid desaturases, transcription factors, protein kinases, cytochromes, etc.) that are highly associated with peach fruit volatiles, and which could prove useful in breeding or for biotechnological purposes.

## Background

Peach (*Prunus persica* L. Batsch) was definitively positioned as a new fruit model when its genome was sequenced and released in 2010 by an international initiative [1,2]. This is further supported by the availability of several genetic and genomic tools, including molecular markers, genetics maps, transient fruit expression assays, microarrays, EST databases, and a 9K SNP array [1,3-7], and by the fact that peach fruit is a drupe and therefore has a different physiology, anatomy, and metabolism from other “post-genomic” fruit crops, such as grape and tomato (berries), strawberry (an aggregate of achenes), orange (speridium), and apple (a pome-type fruit). Hence, the peach represents an excellent opportunity to isolate novel genes related to specific traits, like aroma volatiles. Nevertheless, there still exist many obstacles to discovering gene function in peach. For example, *in-silico* analyses are still quite restricted when compared to other species, like *Arabidopsis,* for which more exhaustive phenotypic and molecular database repositories are available. The use of *a priori* knowledge (e.g., based on co-expression data) to select genes for functional analyses is very limited in peach, which makes it necessary to develop in-house data in order to identify the candidate genes associated with important traits or physiological processes.

Underlying its emerging role as a fruit model is the fact that peach is an important food commodity with an estimated net worldwide production of 11 billion US$ [8]. Aroma is one of the main attributes that affects fruit quality [9] and has been recognized as one of the main factors that affect peach prices in the market [10]. For this reason, volatile organic compounds (VOCs), which define aroma and, in combination with sugars and organic acids, also contribute to fruit taste, have received a great deal of attention. More than 100 VOCs have been described in peach to date ( [11] and references therein), of which about 25 of them appear to conform the typical peach aroma. In particular, γ- and δ-decalactone play a key role in association with C6 compounds, alcohols, esters, terpenoids, and phenolic volatiles [12]. In addition to their contribution to fruit quality, peach volatiles are also important in the food and fragrance industry, where they are used as flavoring agents. Indeed, γ-decalactone is a sought-after industrial product that confers a “peach-like” odor [13] with an expanding annual world demand estimated at 10,000 Kg in 1997 [14]. Despite the importance of lactones, their biosynthetic pathways in peach, and in plants in general, are still poorly understood [15]. An early study suggested that epoxide hydrolases were involved in lactone production, since it was observed that nectarines (a glabrous mutation of peach) are able to produce an artificial lactone when infiltrated with a synthetic, radiolabeled epoxy acid [16]. The analysis of EST libraries later showed that a homologous gene to epoxyde hydrolases was expressed in peach skin [17], although this gene has not been further characterized. Indeed, no gene involved in volatile production in peach has been reported to date. Most studies on genes related to peach aroma have focused on analyzing genes whose homologs are characterized in other plant species, i.e., literature-derived candidate genes. For example, Vecchietti et al. [17] analyzed an EST library to show that a set of candidate genes was expressed in peach fruit and could thus be related to the formation of different volatile compounds. Another study targeted certain members of the carotenoid cleavage dioxigenase gene family for an expression analysis of genotypes differing in carotenoid accumulation to support their involvement in the production of norisoprenoid volatiles in peach [18]. The identification of QTLs for volatile compounds of peach was recently reported [19]. The study in question also proposed putative candidate genes for minor contributors to peach aroma, such as linalool, *p*-menth-1-al, and nonanal, based on low-resolution co-localization of candidate genes within major QTL regions for these volatiles compounds [19]. The use of “omics” technologies is needed to increase the resolution and accuracy of candidate-gene approaches in peach. Biosynthetic pathways for the main volatile compounds in peach are still poorly understood [15], and given the peach-specific nature of the volatiles involved, information from other model species may not be sufficient.

Integrating transcriptomic and metabolomic data by means of pair-wise correlation analyses is emerging as a promising approach for discovering novel gene functions, despite its currently being limited to the model plant *Arabidopsis thaliana* (reviewed in [20]) and tomato [21,22]. On the other hand, integrative “omics” approaches have been widely employed for the purpose of studying diverse physiological processes of plants [23-27] rather than discovering candidate genes.

In order to identify the key genes underlying the production of aroma compounds in peach, we conducted an integrative analysis using in-house-developed transcriptomic and metabolomic data sets. Our reductionist approach, based on correlation analysis [Hierarchical Cluster Analysis (HCA), Pearson Correlation (PC), and Correlation Network Analysis (CNA)] between gene expression and metabolite accumulation levels, allowed us to propose candidate genes for aroma control in peach. Moreover, we cloned and expressed in yeast one of the candidate genes identified herein to analyze its enzymatic activity. Our results demonstrate that the encoded protein possesses ω-6 Oleate desaturase activity, which might participate in the biosynthesis of esters and lactones in peach fruit by generating a common precursor, linoleic acid. In addiction, our results provide some insights into the physiology and metabolism of peach, which are discussed. To the best of our knowledge, this is the first study to use non-targeted approaches to identify candidate genes in peach. The set of genes related to aroma compounds provided herein could prove useful for marker-assisted breeding and/or biotechnology improvements of peach fruit.

## Methods

### Fruit material and analysis

Peach fruit (genotypes ‘MxR_01’ and ‘Granada’) were harvested in July 2009 from a local commercial orchard situated in Murcia, Spain. ‘MxR_01’ is a freestone melting-flesh peach which was obtained through the IVIA (*Instituto Valenciano de Investigaciones Agrarias*) breeding program. ‘MxR_01’ is a seedling from the cross between ‘RedCandem’ and ‘Maruja’, a traditional Spanish germplasm. ‘Granada’ is a clingstone non-melting peach low chilling obtained from a Brazilian breeding program. Fruits corresponding to four maturity stages (S1, S2, S3, and S4) were harvested from one tree per genotype. For S1, S2, and S3, ten fruits from each maturity stage were collected and maturity parameters were immediately analyzed. For S4, “harvest ripe” (or ′ready to buy′) stage, 20 fruits were harvested and divided into two groups. One group (denominated S4) was immediately analyzed and the other (denominated S4+SL) was subjected to shelf-life conditions (2 days at 20°C, 85% RH) prior to the maturity analysis. S4+SL corresponded to “consumption ripe” (or “ready to eat”) stage. The maturity parameters (external color, flesh firmness, weight, total soluble solids (SSC), and ethylene and CO_2_ production) were analyzed as described in Sanchez et al. [11]. A Principal Component Analysis was carried out with maturity parameters in order to identify the three most homogeneous fruits per maturity stage for each genotype (data not shown). These three fruits (biological replicates) were selected for volatile and microarray analyses.

### Sample preparation and HS-SPME-GC-MS conditions

Frozen samples of fruit mesocarp were ground to powder in liquid nitrogen and used for volatile and microarray analyses as follows. Volatile compounds were analyzed from 500 mg of frozen tissue powder, as previously described [11]. The volatile analysis was performed on an Agilent 6890N gas chromatograph coupled to a 5975B Inert XL MSD mass spectrometer (Agilent Technologies). For the chromatography and mass spectra conditions, see Sánchez et al. [11]. A total of 36 commercial standards were used to confirm compound annotation; the VOCs confirmed are listed in Additional file 1: Figure S1. Volatiles were quantified relatively by means of the Multivariate Mass Spectra Reconstruction (MMSR) approach developed by Tikunov et al. [28]. A detailed description of the quantification procedure is provided in Sánchez et al. [11].

### RNA extraction

RNA was extracted from 3 g of frozen tissue powder as described by Meisel et al. [29]. RNA quantity and purity were determined spectrophotometrically with Nanodrop (Nanodrop Technologies Inc.; http://www.nanodrop.com/). RNA integrity was verified by agarose gel electrophoresis. After the quantity and quality checks, RNA were used for the microarray analyses and the qRT-PCR as described below.

### Microarray hybridization and scanning

The microarray analysis was performed essentially as described in Ogundiwin et al. [5]. For the microarray hybridization, the RNA from the samples and the reference pool (1μg of each sample) were amplified and aminoallyl-labeled using the MessageAmp™ II aRNA Kit (Ambion, http://www.ambion.com) and 5-(3-aminoallyl)-20-deoxyuridine-50-triphosphate (aa-dUTP, Ambion), following the manufacturer’s instructions. For each RNA sample, 7.5 μg of aminoallyl-labeled aRNA were re-suspended in 0.1 M Na2CO3 (pH 9.0) and labeled with either Cy5 or Cy3 Mono NHS Ester (CyTM Dye Postlabelling Reactive Dye Pack, Amersham), respectively. Samples were purified with MegaclearTM (Ambion) following the manufacturer’s instructions. Incorporation of Cy5 and Cy3 into probes was measured with a Nanodrop spectrofluorometer (Nanodrop Technologies Inc., http://www.nanodrop.com/). Microarray hybridization of samples and references to the ChillPeach microarray slides was performed manually using Telechem Hybridization Chambers (Corning), following the manufacturer’s instructions. After hybridization, slides were washed in 2x SSC, 0.1% SDS for 5 min at 42°C, 0.1x SSC, 0.1% SDS for 10 min at room temperature, 0.1x SSC for 5 min at room temperature 4 times, and 0.01x SSC for 5 min at room temperature 4 times. Arrays were drained by centrifugation at 528g for 2 min. Slides were scanned with a GenePix 4000B scanner (Axon Instruments) at 10 μm resolution, 100% laser power and with different PMT values to adjust the ratio to 1.0. Microarray images were analyzed and globally normalized using the GenePix 4.1 software (Axon Instruments). Only the spots with a background-subtracted intensity greater than 2-fold the mean background intensity in at least one channel were selected for the analysis. Data files were imported into Acuity 4.0 (Axon Instruments) and normalized by the Lowess normalization method. Finally, only the spots with valid values in at least 2 of the 3 analyzed hybridizations were considered for further analysis. The means and standard deviations of the values were calculated from each sample as log2 values and were later normalized to the median of the reference pool.

### Real-time qRT-PCR analysis

One microgram of total RNA was used to synthesize first-strand cDNA using the SuperScript first-strand synthesis system for RT-PCR (Invitrogen). Two microliters of diluted cDNA (100 ng/μL) were used for qRT-PCR using the SYBR Green PCR master mix (Applied Biosystems), following the manufacturer’s recommendations, and an ABI Prism 7000 sequence detection system (Applied Biosystems). Each biological replicate was assayed in triplicate. Gene-specific oligonucleotide primers were designed using the Primer Express_ version 2.0 software (Applied Biosystems). Primer information is available in Additional file 2: Table S1. The expression levels for target genes were calculated in relation to a reference gene by the DDthreshold cycle method (DDCt, Applied Biosystems). From the microarray data, the gene (ID: PPN078E12, http://bioinfo.ibmcp.upv.es/genomics/ChillPeachDB), whose profile was corroborated as being constant throughout both time courses, was selected as a reference for the normalization of all the subsequent qRT-PCR analyses. The relative gene expression for each candidate gene was expressed by means of the DDCT method using the expression value of the reference gene to normalize expression and the sample from stage S1 in each time-course series as reference samples.

### Data analysis

The Acuity 4.0 software (Axon Instruments) was used for: hierarchical cluster analysis, heatmap visualization, principal component analysis, Pearson correlation evaluation, and for the Student’s t-test for significant differences of volatile levels.

To detect differentially expressed genes between fruit at harvest (S4) and after shelf-life simulation (S4+SL) in both genotypes, data were analyzed with the SAM (Significance Analysis of Microarray) package [30]. Statistical significance was assessed using a two-class (unpaired) SAM analysis, with a false discovery rate of 5% and a q-value of < 0.05.

Correlation network analyses were conducted with the Expression Correlation ( http://www.baderlab.org/Software/ExpressionCorrelation) plug-in for the Cytoscape software [31]. Network topological parameters were calculated with the NetworkAnalyzer plug-in [32]. Networks were visualized with the Cytoscape software, v2.8.2 ( http://www.cytoscape.org).

Venn diagrams were drawn with Microsoft PowerPoint.

### Cloning and bioinformatics analysis of the peach candidate gene

The cDNA (from ‘Granada’ fruit at S4+SL) synthesized by qRT-PCR was used as a template for cloning the ORF of candidate gene PP1002E07. Coding sequences were amplified by PCR (FAD_F: ATGGGTGCCGGTGGAAGAAT and FAD_R: TTATAACTTATTATTGTACC) using Taq DNA polymerase (Biotools B and M Labs SA, Spain). PCR products were cloned in pCR®8 TOPO (Invitrogen, Spain), according to the manufacturer’s instructions, in order to create the pEntry-PpFAD_1B-6 vector. Cloned ORFs were verified by sequencing both DNA strands.

For the sequence analyses, different tools were used. Protein sequences were aligned by the clustalW method using the MegAlign software (DNAStar). Transmembrane domains were predicted with TMpred ( http://www.ch.embnet.org) and protein localization with ProtComp v9.0 ( http://linux1.softberry.com/berry.phtml).

For yeast expression vector construction, the coding sequence was PCR-amplified from the pEntry-PpFAD_1B-6 vector with forward primer FAD-Kozack_F (GGCATGGGTGCCGGTGGAAGAAT) and reverse primer FAD_R. FAD-Kozack_F includes three non-template nucleotides at the 5′ end (underlined) to improve protein synthesis in yeast. The PCR product was cloned in pYES2.1 TOPO® vectors (Invitrogen) by TA cloning following the manufacturer’s instructions. After checking the insert by sequencing, the plasmid was introduced into *Saccharomyces cerevisiae* strain W303-1A MATa [leu2-3112 trp1-1 can1-100 ura3-1 ade2-1 his3-11,15} for expression analysis as follows.

### Expression of the peach candidate gene in yeast and fatty acid analysis

Yeast cells were grown on a rotary shaker at 200 rpm at 30°C in synthetic defined (SD) medium containing raffinose as a carbon source and mixtures of amino acids and nucleoside precursors for marker selection. The expression of the transgenes was induced by the addition of galactose to 2% (w/v) or 1% glucose as a negative control. After 3 days of induction at 20°C, cells were collected, washed with water, and dried. Fatty acids were extracted and methylated from cell pellets and the latter were silanized to obtain trimethylsilyl derivatives, as previously described [33]. The methyl-ester derivatives were analyzed by gas chromatography (GC) with a Hewlett–Packard 6890 gas chromatograph (Palo Alto, CA, USA). The column, chromatographic, and detection conditions are described in Venegas-Calerón et al. [33].

## Results

As part of our final goal of identifying genes and sources of variability to improve peach quality, we undertook complementary genetics and genomics approaches. ‘MxR_01’ and ‘Granada’ peach genotypes vary greatly for important traits, such as melting/non-melting, freestone/clingstone, chilling requirement, aromas, and fruit flavour. To exploit this variability, an F1 population was developed to analyze quantitative trait loci, which will be presented elsewhere. Here we present together the analyses of gene expression and volatile accumulation during fruit maturity and ripening of fruit of the parental genotypes in order to find aroma-related genes.

### Physiological and shelf-life ripening of peach genotypes

In order to characterize the ripening stages of the ‘Granada’ and ‘MxR_01’ peach genotypes, typical maturity parameters were evaluated and are presented in the supplementary data (Additional file 3: Figure S2). The results indicate that, as expected, peel ground color and weight increased with fruit ripening, whereas flesh firmness decreased, but with differences between varieties. Soluble solids content (SSC) was not affected throughout the study period, which indicates that this parameter is not a suitable indicator of ripening for either of the genotypes analyzed. Ethylene and CO_2_ production was also monitored, as the evolution of these compounds reflects the physiological ripening stage of fruits and could also underlie the differences observed in ripening. Both genotypes, ‘Granada’ and ‘MxR_01’, showed increased ethylene production at mature stages (S3 for ‘Granada’ and S4 for ‘MxR_01’), which is typical of climacteric fruit with differences in degrees (Additional file 3: Figure S2). Shelf-life ripening was also used to increase the complexity of our data set and to evaluate the effect on volatile and gene expression. Storage at 20°C for 2 days affected peel ground color, firmness, and ethylene production in both genotypes (Additional file 3: Figure S2). CO_2_ production decreased in both genotypes with shelf-life simulation, although differences were only significant for the ’MxR_01’ genotype. These results indicate that our postharvest treatment was effective in stimulating ripening off the tree in the fruit of both genotypes and that, all together, our samples represent different stages of fruit development and ripening that can be interrogated to obtain the network of interactions between transcripts and volatiles.

### Non-melting (‘Granada’ genotype) and melting (‘MxR_01’ genotype) peaches showed different volatile evolution patterns during ripening

**Figure 1:**
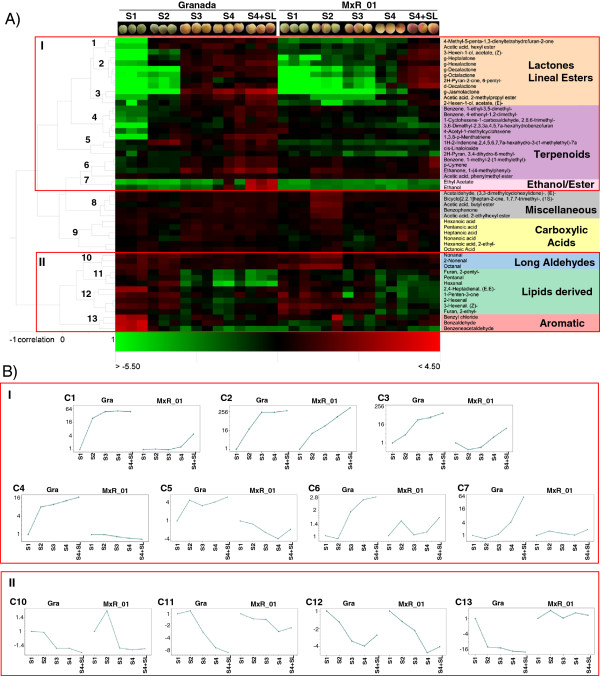
**Volatile evolution patterns in the ‘Granada’ (Gra) and ‘MxR_01’ time-course series. A)** The heatmap and cluster analysis of volatile compounds in the two time-course series (three replicates per stage are shown). The clusters observed (1–13) are indicated in the dendrogram. Data are expressed as log2 of a ratio (sample/common reference). Red boxes indicate those groups of compounds that increase (I) or decrease (II) during ripening. **B)** The evolution patterns for volatiles that change during ripening (I and II). An average profile of all the members of each cluster is shown. For each maturity stage (S1 to S4) and shelf-life response (S4+SL), the mean of the samples (n=3) is shown. Data are expressed as fold changes in relation to S1 (of each genotype) on the log2 scale. The positive region of the y axis is used for values higher than those of S1, while the negative region is for values that are lower than those of S1.

A total of 52 volatile compounds, most of which contribute to peach aroma, were profiled in the fruit samples of the ‘Granada’ and ‘MxR_01’ genotypes at the different ripening stages and at postharvest treatment. The odor descriptors for the analyzed volatile compounds are provided in the supplementary data (Additional file 1: Figure S1). A heatmap and cluster analysis of the volatiles in the two time-course series are shown (Figure 1A). Different trends in volatile evolution can be readily discovered by a simple inspection of the heatmap: the levels of some volatiles increased during ripening (Group I), while others decreased (Group II). Moreover, some compounds, i.e., those belonging to cluster (C) 8, exhibited high levels at specific maturity stages. In addition, some compounds displayed no specific trend during either time series (C9). Several volatile evolution patterns were identified for the compounds that showed increasing or decreasing levels during ripening (Figure 1B), and compounds often grouped according to known biochemical pathways and/or chemical structure (Figure 1A).

Clusters C1, C2, and C3 are formed by lactones and some lineal esters (Figure 1A). They showed similar overall trends in which compound levels increased in both genotypes, but with different profiles and fold changes (Figure 1B). In ‘Granada’, the volatiles in these clusters reached high levels at S3 and remained high for the remaining time series (mainly for C1 and C2), while in ‘MxR_01’, the increment observed throughout the time-course series was gradual (mainly for C2 and C3).

Clusters C4, C5, and C6 are formed mostly by terpenoid volatiles. They increased during ripening in ‘Granada’, while in ’MxR_01’ they showed no changes, or even decreased (C5) during ripening.

Ethanol and Ethyl acetate (C7) levels rose at a mature stage (S4) and even reached higher levels with shelf-life conditioning (64-fold as compared to S1) for ‘Granada’, while in ‘MxR_01’, its content increased only after shelf life reached a 2-fold difference as compared to S1.

Volatiles that decreased during ripening (II) also showed different evolution profiles according to genotype (Figure 1B). Cluster C10 is composed of aldehydes with eight and nine carbons (Octanal, Nonanal, and 2-Nonenal), which showed moderate changes during ripening (up to 1.5-fold changes in relation to S1, Figure 1B). Clusters C11 and C12 are formed by the volatiles derived from the catabolism of linoleic and linolenic acids, the so-called “green compounds” (Figure 1A). Both clusters displayed a similar decreasing trend in both time-course series (Figure 1B). The phenolic volatiles Benzyl chloride, Benzaldehyde, and Benzeneacetaldehyde (C13) decreased from S1 to S2, and remained at low levels until the end of the time series for ‘Granada’, while they showed no significant changes in the ‘MxR_01’ time series.

Several aroma-related volatiles accumulated at different levels in ‘Granada’ and ‘MxR_01’ when fruits reached the commercial mature stage (S4), which means that they could display very marked differences in aroma. As shown in the supplementary data (Additional file 4: Figure S3), 13 volatiles showed significant differences, with most (12) exhibiting higher levels in ‘Granada’ (the more aromatic variety). Compound γ-jasmolactone, which has a characteristic peach-like odor, showed a 39-fold higher level in ‘Granada’ when compared to ‘MxR_01’. Other compounds with pleasant aroma descriptions included 2-Hexen-1-ol, acetate (E) (fruity), γ-Hexalactone (coconut), and 3-Hexen-1-ol acetate (Z) (fruity), which also had higher levels (between 3-fold and 5-fold) in ‘Granada’ compared to ‘MxR_01’. The only compound with higher levels in ‘MxR_01’ at S4 was Benzeneacetaldehyde (4-fold higher than ‘Granada’ at S4), which has been described as confering a “green” aroma.

### The ‘MxR_01’ genotype showed an enhanced shelf-life response

Due to commercial considerations (handling, long-distance transport, storage, etc.), peaches are generally harvested before their complete maturity, but the subsequent shelf life allows the fruit to ripen to the minimum quality threshold for consumers’ acceptance. To analyze the shelf-life response of the ‘MxR_01’ and ‘Granada’ genotypes, we compared the changes in volatile content and gene expression.

**Figure 2:**
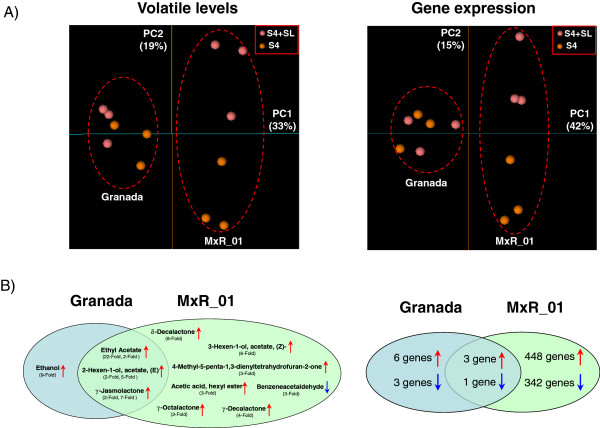
**Response of the ‘Granada’ and ‘MxR_01’ genotypes to shelf-life ripening. A)** A principal component analysis of the volatile compounds (left) and the gene expression (right) data sets. The first two principal components are shown (PC1 and PC2) for each analysis. Three replicates per genotype and treatment are shown. The red dotted circles indicate the genotype of the samples. **B)** Venn diagram of volatiles (left) and genes (right) that change significantly (p<0.05 for the volatile data, and FDR and a q-value of <0.05 for the gene expression data) after shelf-life ripening. Red and blue arrows indicate that the volatile or gene expression increase or decrease, respectively, after treatment. For volatiles, the fold change is indicated in parentheses below each one. For the three compounds that change in both genotypes, the fold change for ‘Granada’ is indicated on the left, while that for ‘MxR_01’ is on the right. For genes, the number of genes increasing or decreasing after treatment is indicated.

Interestingly, the ‘MxR_01’ genotype showed a much enhanced volatile response as compared to the ‘Granada’ genotype when fruit were ripened under shelf-life conditions (Figure 2). The principal component analysis of our volatile analysis of peach samples showed that the second principal component (explaining 19% of variance) clearly separated the fruit samples that were, or were not, subjected to shelf-life treatment in the case of ‘MxR_01’ (Figure 2A, left). However, the ‘Granada’ samples (S4 and S4+SL) were close to each other in the first two components (Figure 2A, left), indicating a weaker postharvest response in relation to the response of ‘MxR_01’. Principal component 1 explained 33% of total variance and mainly separated fruit samples according to differences in genotype (Figure 2A, left). To graphically summarize how shelf-life simulation affects volatile production in both genotypes, a Venn diagram is shown in Figure 2B. For ‘MxR_01’, 10 volatiles showed significant differences (levels of 9 of the 10 volatiles increased) after shelf life, while for ‘Granada’, the levels of only four volatiles changed significantly (once again, they all increased) after shelf-life simulation (Figure 2B, left). As expected, all the volatiles with increased levels after treatment in both genotypes have been described to impact the aroma of mature fruit (for a description of the aroma of these volatiles, see Additional file 1: Figure S1). Accordingly, the levels of Benzeneacetaldehyde, which is associated with the aroma of immature fruit, decreased after postharvest treatment in ‘MxR_01’. As a result, with shelf-life simulation (S4+SL), the initially flat-odor ‘MxR_01’ reached higher levels than ‘Granada’ (at S4+SL) for certain pleasant volatiles. This is the case of 2H-Pyran-2-one 6-pentyl, δ-Decalactone, 3-Hexen-1-ol acetate (Z)-, γ-Decalactone, γ-Octalactone, and γ-Heptalatone (Additional file 5: Table S2). On the other hand, ‘Granada’ still had higher levels than ‘MxR_01’ for other mature fruit-related volatiles (Ethyl Acetate, γ-Jasmolactone, Acetic acid 2-methylpropyl ester, γ-Hexalactone, 2-Hexen-1-ol acetate (E), and Acetic acid butyl ester). Neither of the two genotypes showed a predominance of immature-related volatiles in the mature ripening stages, although some differences were detected between cultivars. While ‘Granada’ showed higher levels of 2,4-Heptadienal, (E,E)-, 1-Penten-3-one, and 2-Hexenal, ‘MxR_01’ displayed higher levels of Furan, 2-pentyl-, and Hexanal (Additional file 5: Table S2). Moreover, ‘Granada’ at S4+SL showed higher levels for some terpenoid compounds (1,3,8-p-Menthatriene, cis-Linaloloxide, and 4-Acetyl-1-methylcyclohexene) than ‘MxR_01’ (Additional file 5: Table S2).

To analyze the effect of shelf-life treatment on the transcriptome, we conducted a PCA analysis with the 4348-gene data set and studied the differentially expressed genes between S4 and S4+SL in both genotypes. The first component (PC1, explaining 42% of variance) separated samples according to genotype (Figure 2A, right). The second component (PC2) explained 15% of variance and separated between the S4 and S4+SL replicates of the ‘MxR_01’ genotypes. While the ‘Granada’ samples remained close together in the first 2 components space, the ‘MxR_01’ samples separated according to treatment (Figure 2A, right), indicating that shelf-life has a greater impact on gene expression in the ‘MxR_01’ genotype than in the ‘Granada’ one. Accordingly, the direct analysis of the differentially expressed genes between S4 and S4+SL in ‘Granada’ and ‘MxR_01’ revealed a stronger gene expression response to the shelf-life in the ‘MxR_01’ genotype (Figure 2B, right). By taking a False Discovery Rate (FDR) and a q-value of < 0.05 as criteria, we found that for ‘Granada’, 13 genes were differentially expressed after treatment (nine up-regulated and four down-regulated), while the ‘MxR_01’ genotype fruits showed a drastic change in gene expression after shelf-life simulation, with 794 differentially expressed genes (451 up-regulated and 343 down-regulated). Only four genes (three up- and one down-regulated) showed the same trend in both genotypes (Figure 2B, right, Additional file 6: Table S3). The gene showing the largest changes after shelf-life in both genotypes (id: PPN044F04) had a calcium-binding EF hand domain. A gene related to the metabolism of amino acids (Aspartate aminotransferase) showed moderate changes in both genotypes, increasing after treatment. The third gene which increased after shelf-life (id: PPN064C01) is a homolog to a translation initiation factor, suggesting that protein synthesis could be stimulated by treatment in both genotypes. Only one gene decreased after shelf-life in both genotypes, an acid phosphatase belonging to class B. The ‘MxR_01’ genotype showed a higher decrease in the expression of this gene (3.2-fold) compared to ‘Granada’ (1.8-fold).

These results support that mostly ‘MxR_01’ fruits undergo significant molecular, and consequently physiological, changes during postharvest conditioning, and that very little happens to ‘Granada’. It is worth mentioning that despite the larger number of genes showing significant differences in expression after shelf-life in ‘MxR_01’ (794 genes vs. 13 genes in ‘Granada’), most involved only minor fold-change differences. Of the genes, 9% (74:794) showed a fold change of over 2 (between S4 and S4+SL) for the MxR genotype. For ‘Granada’, nine of the 13 differentially expressed genes showed fold differences over 2. In the supplementary data (Additional file 7: Table S4), a complete description of differentially expressed genes with a 2-fold change cutoff is provided.

### Identification of the genes related to peach volatile compounds by hierarchical cluster analyses

In order to obtain a global view of the interrelationships between volatile accumulation and gene expression, a Pearson correlation analysis was conducted for all the possible volatile gene pairs (Additional file 8: Table S5). Volatiles showed wide variability in the range of the distribution of volatile-gene correlations (data not shown). For example, Hexanoic acid 2-ethyl- had correlations ranging from −0.42 to 0.52, while γ-Jasmolactone showed correlations ranging from −0.92 to 0.96. Thus, our data set contains some volatiles which have no clearly associated genes, while others show many strongly correlated genes.

**Figure 3:**
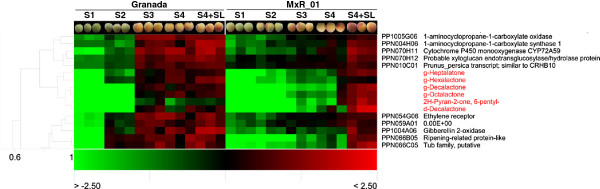
**Hierarchical cluster analysis for indentifying correlated genes.** Detail of the heatmap and cluster analyses where C2 lactones (in red) are found. For each gene, the id and the unigene annotation are provided. Three replicates per maturity stage are shown. Data are expressed as a log2 of a ratio (sample/common reference). The gene annotated as “0.00E+00” indicate that either no homologue was found or the homologue found has unknown function. For a detailed description of ChillPeach unigene functional annotation see Ogundiwin et al. [5].

In order to identify the genes related to a family of co-regulated volatiles, a Hierarchical Cluster Analysis (HCA) was conducted with the complete data set (4348 genes and 52 volatiles × 30 samples). The Pearson correlation coefficient was used as the similarity metric and a complete linkage method was analyzed in order to find the variables (i.e., VOCs or genes) correlating with all the members of each cluster. As shown in Additional file 9: Figure S4B, most of the volatiles remained associated with the same cluster members, as previously described in Figure 1 (clusters C2, C5, C6, C7, C8, C9, C10, and C11), while other metabolites (those belonging to clusters C1, C3, C4, C12, and C13) correlated better with transcripts as compared to other volatiles and formed new clusters. The metabolites belonging to clusters C6, C8, C9, C10, and C11 correlated poorly to transcripts in the HCA, as the correlation values with gene clusters were below 0.6. Other volatiles (belonging to C2, C5, and C7) fall into the same cluster, and correlate well with some genes (implying higher cluster correlations than 0.6). For the volatiles in cluster C4, four genes (PP1009D02, PPN027C05, PPN063A04, and PPN069C04) were found in the same sub-cluster, indicating a good association of these genes with all of these volatiles. In contrast, the volatiles in clusters C1, C3, and C13 (according to Figure 1) correlated better with certain transcripts than with other volatile members of the cluster. Based on these results, a series of genes corresponding to transcripts whose levels were highly correlated to volatile levels was selected. A list and short description of the selected genes is provided in Table 1, with further details provided in the supplementary data (Additional file 10: Table S6). Certain genes, despite their being present in the same cluster, were not selected as candidate genes for the associated volatile because their functional annotation suggested that the genes could affect general ripening. For example, all the members of cluster C2 are lactones and were associated with a cluster of five genes (Figure 3). Two of them are genes relating to ethylene biosynthesis (PP1005G06 and PPN004H06) and another is related to cell wall softening (PPN070H12). Even though this group of volatiles and genes shared a common regulation (i.e., the ripening process), they are clearly not good candidates for the specific control of lactone accumulation. They are probably associated with a general control of other ripening aspects and were, therefore, not selected in Table 1. A transcription factor with no homologs in *Arabidopsis* (PPN010C01) and a cytochrome P450 monooxygenase (PPN070H11), which is probably involved in lipid metabolism, were selected as putative candidates.

**Table 1:** Genes that highly correlate with volatile compounds proposed as candidate genes

**Genes for volatile cluster C1**			
**id**	**Unigene annotation**	**Identified by**	**Match**
PPN009B08	Protein At1g45233	HCA, CNA	ppa001502 m
PPN078H01	Acid phosphatase 1 precursor (EC 3.1.3.2) (Apase-1(1))	HCA	ppa010063 m
PP1009A02	CAMP response element binding (CREB) protein	HCA, CNA	ppa012739 m
PPN001H09	YUP8H12R.39 protein	HCA, CNA	ppa005452 m
PPN066C05	Tub family, putative	CNA	ppa006121 m
PPN066B05	Ripening-related protein-like	CNA	ppa011478 m
**Genes for volatile cluster C2**			
**id**	**Unigene annotation**	**Identified by**	**Match**
PPN010C01	Prunus_persica transcript; similar to CRHB10 [Ceratopteris richardii (Triangle waterfern)]	HCA	ppa008984 m
PPN070H11	Cytochrome P450 monooxygenase CYP72A59	HCA, PC, CNA	ppa006310 m
PPN059A01	0.00E+00	PC	ppa013582 m
PPN026D01	no_annotation_available	PC, CNA	ppa009231 m
PPN037A04	Phosphatidylinositol transfer protein/retinal degeneration b protein	HCA, PC	ppa010020 m
**Genes for volatile cluster C3**			
**id**	**Unigene annotation**	**Identified by**	**Match**
PP1002E07	Omega-6 fatty acid desaturase	HCA	ppa007098 m
PP1001A05	Pyruvate decarboxylase 1	HCA	ppa003086 m
PP1004F06	Vesicle-associated membrane protein 727	HCA	ppa010737 m
PP1004G05	no_annotation_available	HCA, CNA	ppa004658 m
PP1004H08	no_annotation_available	HCA	ppa004582 m
PP1005B05	AT4g00090/F6N15_8	HCA	ppa004482 m
PPN009C07	AJ533535 S3II Prunus persica cDNA clone PP_S3II_T5_SP6, mRNA sequence	HCA	ppa004933 m
PPN010B11	Serine-threonine protein kinase, putative	HCA, PC, CNA	ppa008251 m
PPN070B11	Kinase-like protein	HCA	ppa003659 m
PPN070D11	Protein At5g25010	HCA	ppa009131 m
PPN075B07	F8K7.22 protein	HCA	ppa008595 m
PPN032F06	PDR-like ABC-transporter	HCA, PC, CNA	ppa000267 m
PPN036E10	At5g03345	HCA, CNA	ppa015891 m
PPN069F09	PK11-C1	HCA, PC, CNA	ppa006108 m
PPN002B03	Malus_x_domestica transcript; similar to F17O7.2 [Arabidopsis thaliana]	PC	ppa007712 m
PPN031F12	Novel plant SNARE 13	HCA, PC	ppa010000 m
PPN031G12	Os09g0509300 protein	PC, CNA	ppa002575 m
PP1004C02	Calcium binding protein	PC, CNA	ppa011722 m
PPN051H01	At1g72790/F28P22_2	CNA	ppa002494 m
**Genes for volatile cluster C4**			
**id**	**Unigene annotation**	**Identified by**	**Match**
PP1009D02	IAA16 protein	HCA, CNA	ppa011570 m
PPN027C05	PHB2	HCA, CNA	ppa009505 m
PPN063A04	T25K16.5	HCA	ppa011444 m
PP1000F05	Snakin-1	CNA	ppa014086 m
PPN017C08	ECA1 protein	CNA	ppa012387 m
PPN017B08	AT3g05350/T12H1_32	CNA	ppa002173 m
**Genes for volatile cluster C5**			
**id**	**Unigene annotation**	**Identified by**	**Match**
PPN029H12	AJ827262 S3 Prunus persica cDNA clone S312E11, mRNA sequence	HCA, CNA	ppa025397 m
PPN053G07	Putative pod-specific dehydrogenase SAC25	HCA, CNA	ppa008709 m
PPN066C10	Cytochrome P450	HCA	ppa004664 m
PPN078H04	Putative pod-specific dehydrogenase SAC25	HCA, CNA	ppa008713 m
**Genes for volatile cluster C7**			
**id**	**Unigene annotation**	**Identified by**	**Match**
PPN030E11	PP_LEa0004N09f Peach developing fruit mesocarp	HCA	ppa002860 m
PPN071H07	Enolase 1	HCA	ppa005779 m
PPN027H11	Phox-like	HCA	ppa001676 m
**Genes for volatile cluster C11**			
**id**	**Unigene annotation**	**Identified by**	**Match**
PPN052H12	Desaturase delta 9	CNA	ppa009359m
**Genes for volatile cluster C12**			
**id**	**Unigene annotation**	**Identified by**	**Match**
PP1003B11	0.00E+00	HCA	ppa010788 m
PP1009F04	PU1_plate44_F06 PU1 Prunus persica cDNA weakly similar to putative protein (AL161573)	HCA	ppa009611 m
PPN006F05	GTP-binding protein SAR1B	HCA	ppa011838 m
PPN033D11	Putative acid cluster protein 33	HCA	ppa006430 m
**Genes for volatile cluster C13**			
**id**	**Unigene annotation**	**Identified by**	**Match**
PP1001G06	Cysteine protease CP1	HCA	ppa005199 m
PP1004F12	Dehydrin	HCA	ppa005514 m
PPN018G03	Putatative tyrosine aminotransferase	CNA	ppa006368 m

Genes were identified by either: a Hierarchical Cluster Analysis (HCA), a simple Pearson Correlation (PC) analysis and/or a Correlation Network Analysis (CNA). For each gene, microarray id (id), the unigene annotation, and the most similar gene predicted in the peach genome (Match) are shown. The genes annotated as “0.00E+00” indicate that either no homologue was found or the homologue found has unknown function. For a detailed description of ChillPeach unigene functional annotation see Ogundiwin et al. [5].

### Genes related to the main aroma compounds of peach

Although aroma is a complex trait, and each volatile contributes to defining overall peach aroma, some compounds appear to be more important than others according to sensory assessments (Additional file 1: Figure S1). For example, the aroma of two lactones, γ-Decalactone and γ-Jasmolactone, is described as peach-like [34]. We have evaluated correlations between the levels of gene transcripts and the levels of these two aroma volatiles across the different samples in order to find genes related to them that were not identified in the previous analysis. Additional file 11: Table S7 provides the ten genes with the highest direct correlation values; in addition, the full list of genes with correlation values between the corresponding transcript and volatile levels is included in the supplementary data (Additional file 8: Table S5). As expected, some of the genes identified in the HCA were also found in this analysis. For example, genes related to ethylene signaling proved to be highly correlated with γ-decalactone (PPN054G06 and PPN004H06) and γ-Jasmolactone (PP1005G06). Cytochrome P450 (PPN070H11) was strongly correlated with both lactones. γ-Jasmolactone also showed high correlations with a protein involved in metabolite transport (PPN032F06) and with two protein kinases (PPN069F09 and PPN010B11). Nevertheless, two other genes that were not previously identified (PPN059A01 and PPN026D01) with no homology in *Arabidopsis* showed the highest correlations with γ-decalactone (r=0.90 and r=0.87, respectively). Moreover, γ-Jasmolactone was highly correlated with the genes showing no homology with *Arabidopsis* (PPN002B03 and PPN031G12), and which were not identified in previous analyses. Interestingly, some genes involved in endoplasmatic reticulum traffic were also associated with lactone production in peach fruit. A homolog to a Phosphatidylinositol transfer protein (PPN037A04) correlated well with γ-decalactone, while a novel plant SNARE 13 (PPN031F12) highly correlated with γ-Jasmolactone. Finally, γ-Jasmolactone showed a good correlation with the level of a transcript that encodes a protein related to the caleosin family (PP1004C02).

### Correlation network analysis for discovering hub genes

**Figure 4:**
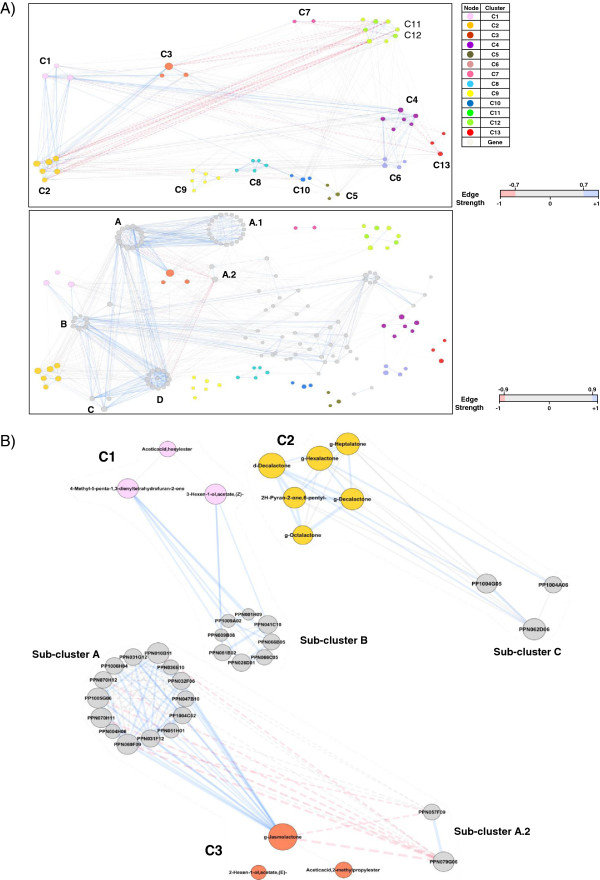
**Correlation network analysis for identifying candidate genes. A)** Network of volatiles (upper) and the integrated network of VOCs and genes (bottom). The nodes representing volatiles are colored according to the cluster that they belong to (C1-C13 according to Figure 1), as indicated. Genes are represented as gray nodes. Edges are colored according to their strength. Note that edges have different codifications for each network, which is indicated at the right of each one. Node size indicates its connectivity; the bigger the node, the higher the connectivity measured as node degree (i.e., the number of edges connecting the node). A, A.1, A.2, B, C, and D indicate the main gene sub-clusters. **B)** Magnification of volatile groups (C1, C2 and C3) showing the interactions with genes in detail. For each gene node, the id is shown. The list of genes belonging to sub-clusters A, A.1, A.2, B, C, and D, as well as some topological parameters, are provided in Additional file 11: Table S7.

A correlation network analysis (CNA) was conducted to further analyze the data and to identify additional genes related to aroma compounds that could have gone unnoticed in previous analyses. The CNA offers several advantages when compared to HCA or Pearson correlation analyses, which evaluate correlations one by one, since it 1) provides a complete view of the interaction between groups of variables and 2) allows the identification of variables (genes or volatiles) that play a central role in the network, the so-called hubs. Nevertheless, as the number of nodes increases, the interactions between them also increase, conceivably causing the network to become incomprehensibly complex at a certain point. To overcome this problem, a new data set comprising those genes with a correlation coefficient higher than 0.85 and with at least one VOC was created. First, a correlation network considering only the 52 VOCs was constructed. As expected, the familiar volatile clustering observed in the HCA (Figure 1) could be readily identified as groups of highly interconnected nodes (Figure 4A, upper) in our CNA. Yet an additional interconnection between those groups emerged. Clusters C1, C2, and C3, all formed by lactones and linear esters, are interconnected by strong direct correlations (r > 0.7). C3 is directly related to Ethanol and its ester (C7) and inversely related to lipid-derived volatiles (C11 and C12). The volatiles of C2 are also inversely correlated with compounds C11 and C12. C1 metabolites are directly related to C4, which, in turn, positively correlates with C6 and negatively correlates with C13. The metabolites belonging to the other clusters (C5, C8, C9, and C10) are interconnected to various other clusters, but show weaker correlations (r < 0.70 in absolute values).

As mentioned previously, in order to analyze interactions with genes, a correlation network was constructed by preselecting those genes with a cutoff of r > 0.85 in absolute values. To integrate the interactions between genes and VOCs, the two networks were merged to create a new one (Figure 4A, bottom). The resulting network consisted of 160 nodes (52 VOCs and 108 genes) and 1364 edges (correlations). The network clearly reflected the different distributions of the correlations between VOCs and genes observed. As the volatiles from C1, C2, and C3 showed the largest number of strong correlations with genes (both positive and negative), their nodes were highly connected to gene nodes (Figure 4A, bottom). Compound γ-jasmolactone showed strong correlations (r > 0.95) with a group of 14 genes (sub-cluster A, Figure 4B). Some members of this sub-cluster had already been selected as candidate genes for the metabolites of C3 (PPN069F09, PPN032F06, PPN031F12, PPN036E10, PPN031G12, PPN010B11, and PP1004C02) or for lactones of C2 (PPN070H11), as described in previous sections. Nevertheless, one gene, which our previous analysis did not reveal, was selected as a candidate gene (PPN051H01, Table 1). Sub-cluster A was related to a group of 20 genes (sub-cluster A.1) by strong positive correlations. It is interesting to note that some of the genes of sub-cluster A are involved in signal transduction processes (Additional file 12: Table S8). For example, there are two putative protein kinases (PPN069F09 and PPN010B11) that show high connectivity (degree=39 and 40, respectively), as well as two ethylene signaling genes (PP1005G06 and PPN004H06). A gene with homology to Asparagine synthetase (PPN079G06) is strongly and inversely correlated with γ-jasmolactone and could be considered a hub given its high connectivity (degree=37, Additional file 12: Table S8, Figure 4B). Volatile cluster C1 was highly related to sub-cluster B by strong correlations with 4-Methyl-5-penta-1,3-dienyltetrahydrofuran-2-one and 3-Hexen-1-ol acetate (Z)- (Figure 4B). Two transcription factors belong to this sub-cluster: one had already been selected (PP1009A02) and the other was a newly identified one (PPN066C05, Table 1). Furthermore, a gene that was not identified previously as showing homology to ripening-related proteins was selected (PPN066B05, Table 1). Sub-cluster B is highly interconnected with sub-cluster C, which consists of three genes with strong correlations with the lactones of C2 (Figure 4B). The members of this sub-cluster are: a gene related to Gibberellin metabolism (Gibberellin 2-oxidase, PP1004A06), a gene that is likely related to cell wall physiology (UDP-arabinose 4-epimerase 1, PPN062D06), and a gene with no homolog in *Arabidopsis* (PP1004G05), which had already been identified (Additional file 12: Table S8). The main sub-clusters that correlated with lactones and esters (A, B, and C) were highly interconnected to a group of 13 genes that formed sub-cluster D (Figure 4A bottom, Additional file 12: Table S8).

With the selected cutoff value (genes with a correlation higher than 0.85), the correlation network analysis failed to identify genes related to the other VOC clusters (C4 to C13). To gain insight into the genes associated with these volatile compounds, a new data set was composed by selecting genes after lowering the cutoff to > 0.8 for volatiles belonging to clusters C4 to C13, which allowed a new correlation network to be constructed (Additional file 13: Figure S5). The aromatic VOCs in C13 are related to a putative tyrosine (Tyr) aminotransferase (PPN018G03) through a direct correlation with Benzeneacetaldehyde (Additional file 13: Figure S5B) and, therefore, was selected as a candidate gene (Table 1). The VOCs from C4 are correlated with a group of 5 genes, two of which are related to hormone signaling. One is an Auxin-responsive protein (IAA16, PP1009D02) and the other (PP1000F05) belongs to a family of proteins regulated by gibberellins. These 5 genes correlated well with a group of 42 genes (sub-cluster E), and some were also associated with auxin (PPN044D01 and PP1005H05) and gibberellin (PPN037E06) signal transduction pathways (Additional file 13: Figure S5A). In more detail, we see that the lipid-derived compounds Furan, 2-pentyl-, and Hexanal inversely correlated with a lipid delta 9 desaturase homolog (PPN052H12), which, in turn, was highly correlated with a gene with no homology in *Arabidopsis* (PPN023E05). A BZIP-like transcription factor (PPN019F11) strongly correlated with PPN023E05 and with some genes of the sub-cluster E. Compound cis-Linaloloxide (belonging to C5) also strongly correlated with a gene that had a short-chain dehydrogenase/reductase (SDR) domain (PPN053G07). This gene formed a sub-cluster with two other genes, one which also had a short-chain dehydrogenase/reductase (SDR) domain (PPN078H04) and the other with no homologs in *Arabidopsis thaliana* (PPN029H12). The previous selection strategy also revealed these three genes as candidates (Table 1).

### Validation of microarray data by qRT-PCR analysis

In order to validate the expression profile of the candidate genes identified by microarray analysis, gene-specific qRT-PCR analyses were conducted. We selected a subgroup of genes that we identified by their association with the main aroma-contributing volatiles, i.e., lactones and esters. Seven genes selected for clusters C1, C2, and C3 (PP1002E07, PPN070H11, PPN001H09, PPN032F06, PPN002B03, PPN059A01, and PPN066B05) were analyzed. Three genes that were inversely correlated with lactones/esters (PP1002D12, PPN079G06, and PPN008D02, Additional file 8: Table S5) were also included to obtain a better validation of our results. The Pearson correlation coefficient between the expression results obtained with both RNA profiling technologies was evaluated for all the genes studied. The ten genes analyzed by transcript-specific qRT-PCR analysis corroborated the microarray analysis, showing correlation coefficients ranging from 0.710 to 0.988 (Additional file 2: Table S1). By way of example, the comparison between the qRT-PCR and the microarray profiles of gene PPN001H09 is shown in Additional file 14: Figure S6.

### The enzyme encoded by candidate gene PP1002E07 displays ω-6 Oleate desaturase activity

**Figure 5:**
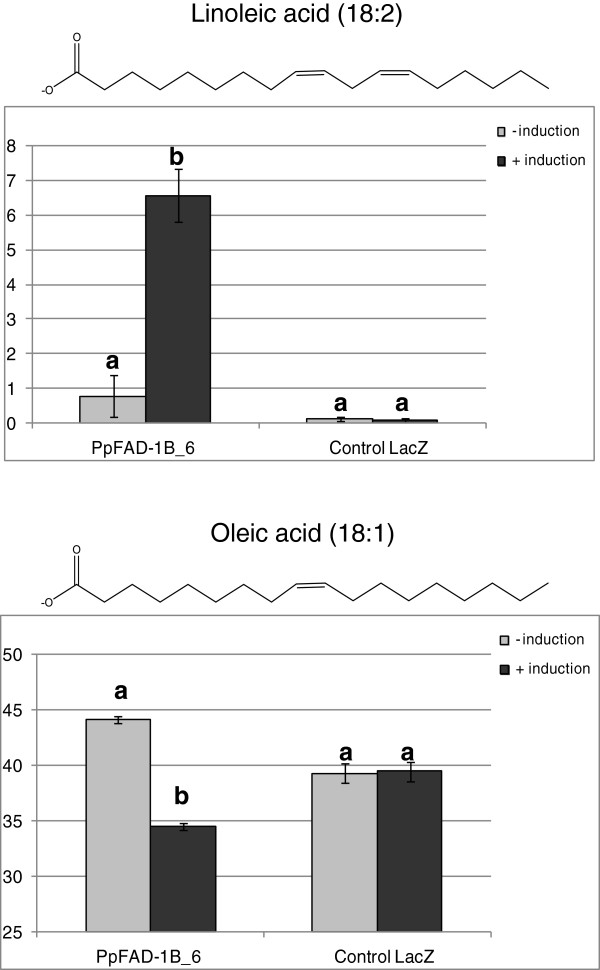
**Content of Linoleic acid (upper) and Oleic acid (bottom) in yeast expressing *PpFAD_1B-6*.** Fatty acid was analyzed in yeast cultures under non-inducing (−) or inducing (+) conditions. Values are provided as a % of total fatty acid content (mean of n=3). Differences between treatments (− induction vs. + induction) were stated by ANOVA (p<0.05) and are indicated with different letters. Error bars indicate standard deviation.

We conducted functional studies for one of the identified candidate genes. The ORF of candidate gene PP1002E07 was amplified by RT-PCR from the RNA of ‘Granada’ fruits at S4+SL and was then cloned. The cloned ORF consisted of 1149 bp and was named *PpFAD_1B-6* (KC169941). The Blastp analysis on the NCBI web revealed that the deduced protein sequence contains the conserved Delta12-FADS-like domain (cd03507), typical of fatty acid desaturases, but also of other FAD-like hydroxylases and epoxidases. The presence of the typical functional elements of FAD-like enzymes was analyzed using bioinformatics tools: six transmembrane domains were identified by TMpred (data not shown) and three His motifs were identified by a sequence alignment of proteins with characterized FAD-Like enzymes (Additional file 15: Figure S7). ProtComp predicted that the PpFAD_1B-6 protein would be localized in the endoplasmatic reticulum (score: 7.81), as is to be expected for this kind of enzyme. To define the enzymatic activity and substrate specificity of PpFAD_1B-6, a DNA fragment encompassing the complete ORF was conditionally expressed in *S. cerevisiae*. Yeast is particularly suitable for this assay as it normally produces neither polyunsaturated nor hydroxylated fatty acids, which are the expected products. The main saturated fatty acids [palmitic acid (16:0), stearic acid (18:0)] and the corresponding unsaturated fatty acids [palmitoleic acid (16:1), oleic acid (18:1), linoleic acid (18:2), and linolenic acid (18:3)], as well as a hydroxylated fatty acid [ricinoleic acid (18:1-OH)] were analyzed. As shown in Figure 5, under inducing conditions, the yeast transformed with the *PpFAD 1B_6* construct produced linoleic acid (18:2) at levels representing up to 6.6% of total fatty acids. Under non-inducing conditions, linoleic acid (18:2) was detected at only trace levels, representing less than 0.8% of total fatty acids, whereas in the control yeast strain (transformed with a vector where the candidate gene was replaced with the LacZ gene), linoleic acid (18:2) was not detected (less than 0.1%± 0.09), indicating that the inducible control was probably leaky. After the induction of *PpFAD 1B_6,* oleic acid (18:1) decreased from 44.2% (±0.3) to 34.5% (±0.3) of the total fatty acids content (Figure 5), indicating that *PpFAD 1B_6* had used endogenous oleic acid as a substrate in the production of linoleic acid and was, therefore, acting as an ω-6 Oleate desaturase. The control strain showed no differences in oleic acid (18:1) levels after induction. Ricinoleic acid (18:1-OH) was not detected in the yeast expressing *PpFAD 1B_6* (Additional file 16: Table S9), suggesting that this enzyme has no Oleate 12-hydroxylase activity. The induction of *PpFAD 1B_6* did not affect the levels of palmitic acid (16:0), estearic acid (18:0), or palmitoleic acid (16:1) (Additional file 12: Table S8), indicating that the encoded enzyme is not capable of introducing a double bond into unsaturated palmitic and oleic acids. Therefore, our results indicate that the candidate gene PP1002E07 encodes functional ω-6 Oleate desaturase activity.

## Discussion

### Non-melting (‘Granada’ genotype) and melting (‘MxR_01’ genotype) peaches show different maturity/ripening and shelf-life responses, which can be exploited to analyze co-regulation patterns

An “omics” approach based on integrative transcriptomics- and metabolomics-derived data was undertaken to identify those genes putatively involved in volatile production in peach fruit. The main aroma-contributing volatiles of peach increase during maturity and ripening following different patterns [35-38]. It is also known that the peach transcriptome undergoes significant re-organization during fruit ripening and maturity [39,40]. Thus, our rationale was that the genes involved in aroma-related volatile production could be discovered by analyzing the co-regulation between gene expression and volatile accumulation during maturity and ripening. In addition, we hypothesized that the inclusion of different peach types could improve the robustness of metabolite-gene relationships since the gene-volatile correlation should hold across the genotype with different ripening characteristics. To this end, four maturity stages were analyzed for clingstone non-melting (‘Granada’) and freestone melting-flesh (‘MxR_01’) peaches (Additional file 3: Figure S2). To add further complexity to the data set and to increase the robustness of our model, one postharvest treatment was also included, since it is known that non-melting and melting peaches respond differently to shelf-life ripening [41]. The volatile profiles analyzed in both time-course series confirmed that these genotypes show a different volatile evolution during maturity and ripening (Figure 1), suggesting that our hypothesis was correct. Each genotype also showed a different response to shelf-life simulation. Volatile profiles were more affected after treatment in ‘MxR_01’ as compared to ‘Granada’, although the lactones and esters in both genotypes increased after treatment (Figure 2), which is in agreement with previous studies [37,38]. Correspondingly, ‘MxR_01’ also showed a more dramatic change in gene expression as compared to ‘Granada’ after the shelf-life simulation (Figure 2), indicating that the restructuration of volatile content after shelf-life could be, at least in part, determined by a modification in gene expression.

Therefore, our approach combined different genetic backgrounds with a development-regulated process and an artificial post-harvest treatment in order to analyze co-regulation patterns. This strategy differs substantially from other previously used integrative approaches. In tomato, the change in gene expression and metabolite accumulation throughout fruit development was also used to identify not only candidate genes, but also variability between different fruit tissues (mesocarp and locular), which was included to improve the robustness of the study [22]. Carrera et al. [21] applied an alternative strategy consisting in profiling (transcripts and metabolites) an RIL population to find candidate genes that affect tomato fruit aroma by modeling the “omics” data obtained. In this case, system perturbation was given by the genomic region introgressed in each line. In other study, *Arabidopsis* plants were starved in order to affect the transcriptome and the metabolome so that they could analyze the co-regulation patterns and thereby identify the genes associated with different metabolites [42,43]. The variation in gene expression and the volatile levels obtained in our study suggest that the strategy used herein could be a suitable alternative for the analysis of co-regulation patterns.

### Lactone and ester production in peach fruit requires a tight regulation of lipid catabolism

A number of candidate genes for peach volatiles were identified using a combination of data analysis techniques based on correlations between gene expression and volatile accumulation data (HCA, PC, and CNA). The functional annotation indicated that, in some cases, this is due to a general effect on ripening. For example, the genes related to ethylene biosynthesis and perception (PPN004H06, PP1005G06, and PPN054G06) directly correlate with lactones (Figure 3, Figure 4, Additional file 11: Table S7), suggesting that they could be regulators of their biosynthesis. It is known that ethylene is the hormone that controls the ripening of climacteric fruits, such as peach [44], which makes it an unsuitable candidate for specific aroma control since its modification could alter the whole ripening syndrome. However, the possibility of there being downstream effectors specific for aroma production, which would be much more ideal candidates, cannot be ruled out, as different aspects of ripening appear to be controlled by specific transduction elements [45]. Overall, these results are not only in agreement with Zhang et al. [46], who indicated that ethylene plays a regulatory role in aroma formation in peach, but also provide additional support for further research to search for the specific ethylene signal transduction elements involved in the specific activation for volatile production during ripening.

**Figure 6:**
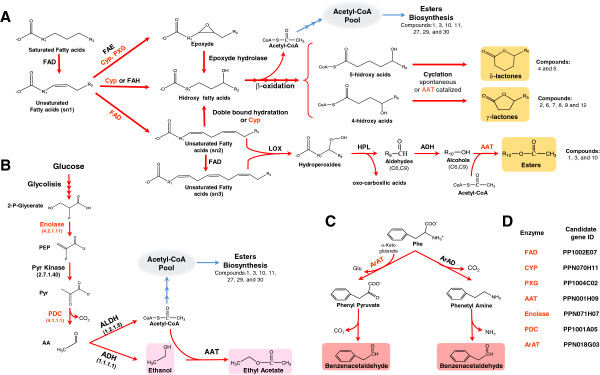
**Proposed models of action for the candidate genes identified. A**) The putative pathways for Lactones and Esters biosynthesis. The δ- and γ-lactones and esters produced (numbered according to Additional file 1: Figure S1) have different alkyl groups at R6, R5, and R10, respectively. C6 and C9 are indicated below the aldehydes and alcohols, which means that the compounds can have six or nine carbons. Please note that different enzymes use free fatty acid or are activated with different groups (CoA, Acyl carrier protein or lipidic group), but must be activated with a CoA group to enter β-oxidation. **B**) Ethanol and Ethyl acetate production. The Acetyl-CoA produced can also be added to the pool that is used to synthesize the numbered esters (according to Additional file 1: Figure S1). **C**) The route for Benzenacetaldehyde biosynthesis in melon (and proposed for peach) and tomato, left and right, respectively. For the three routes (**A**, **B**, and **C**), the enzymes codified by the candidate genes proposed are indicated in red. **D**) The ID of the candidate gene that is proposed for each enzyme. Enzyme abbreviations: FAD, Fatty acid desaturase; FAE, Fatty acid Epoxydase; CYP, Cytochrome P450; PXG, Peroxygenase; FAH, Fatty acid Hydroxylase; LOX, Lipoxygenase; HPL, hydroperoxide lyase; ADH, alcohol dehydrogenase; AAT, Alcohol Acyl Transferase; PDC, Pyr decarboxylase; ALDH, Aldehyde dehydrogenase; ArAT, Aromatic aminoacid Aminotransferase; ArAD, Aromatic amino acid decarboxylase. Compound abbreviations: 2-P-Glycerate, 2-Phospho Glycerate; PEP, Phosphoenol Pyr; Pyr, Pyruvate; AA, Acetaldehyde; Phe, Phenylalanine; Glu, Glutamate.

Volatile clusters C1 and C3 are each formed by two lineal esters and one lactone (which, indeed, are cyclic esters), while the rest of the lactones group in C2 (Figure 1, Additional file 1: Figure S1). All these volatiles accumulated in the fruit during the time-course series in both genotypes, although they trended differently (Figure 1) and are highly interconnected in the correlation network (Figure 3A). Esters are known to be formed by the condensation of an acyl moiety with an alcohol catalyzed by alcohol acyl transferases (AATs) [47]. The structural diversity of esters is mainly due to alcohol, which can originate from different pathways. In this study, the esters found derive from acetyl-CoA and various alcohols, which, in the case of esters 1, 3, and 10 (numbered according to Additional file 1: Figure S1), derive from the lipoxygenase pathway (Figure 6A). In fruits, acetyl-CoA is formed from Pyruvate (Pyr) by the action of two enzymes: Pyruvate decarboxylase (EC 4.1.1.1) and aldehyde dehydrogenase (EC 1.2.1.5, [48]). Accordingly, a Pyruvate decarboxylase associated with ester 2-Hexen-1-ol acetate (E)- was identified by HCA (Additional file 9: Figure S4), and we proposed that Pyr decarboxylase is regulated at the expression level to ensure the supply of acetyl-CoA for aroma volatile ester biosynthesis (Figure 6).

Unlike esters, the lactone biosynthesis pathway in plants is still incomplete. Nevertheless, it seems clear that lactone biosynthesis starts from fatty acids with the introduction of an O atom to form hydroxy fatty acids [15]. Hydroxy fatty acids are then shortened by β-oxidation to form 4- or 5- hydroxy acids, which in turn result in the corresponding lactones after intramolecular esterification (Figure 6A, [15]). However, it is unclear how the introduction of the O atom is achieved. By infiltrating a synthetic radiolabeled epoxy acid into fruits, it has been demonstrated that nectarines (peach glabrous mutants) are able to produce an artificial lactone [16]. Based on this observation, it was proposed that the introduction of the O atom is achieved by fatty acid epoxydation and that the subsequent breakdown of the epoxy group (by epoxyde hydrolase) forms hydroxy acids; unfortunately, no further evidence has been presented. Alternatively, the introduction of hydroxyl groups could be produced by the hydration of unsaturated fatty acids or the direct hydroxylation of fatty acids catalyzed by Cytochrome P450s (CYPs) or by other hydroxylases not related to CYPs (Figure 6A). To date, neither have the proposed endogenous fatty acid modification enzymes been cloned nor has their involvement in lactone biosynthesis in peach fruit been demonstrated.

For volatile cluster C1, an AAT homolog (PPN001H09) was selected as a putative candidate (Table 1, Additional file 10: Table S6) because it is highly associated with the lactone of C1 in the correlation network analysis (Figure 4). Moreover, the expression of this gene is moderately to highly correlated to the other lactones (from r=0.64 to 0.86, Additional file 8: Table S5). It is still unclear whether the last cyclation step leading to lactone biosynthesis requires an enzyme or not [15], but if this were the case, this gene could represent a novel type of AAT that produces cyclic esters (lactones). This AAT (PPN001H09) also correlated with lineal esters (r=0.69 to 0.75, Additional file 8: Table S5), making it therefore possible that this gene encodes a standard AAT. Nonetheless, a phylogenetic analysis has revealed that the AAT found here (PPN001H09) is not related to previously characterized fruit AATs (data not shown). As it is unlikely that this gene can be identified by candidate gene approaches based solely on sequence similarity to the genes reported in the bibliography, these results emphasize the advantage of using non-targeted analyses based on correlation rather than more targeted gene approaches based solely on homology with genes previously described as being associated with a given process. To date, no AAT from peach has been characterized, although an EST analysis has revealed that some AATs are expressed in fruit mesocarp [17]. Moreover, a targeted approach has demonstrated that the expression of another AAT (ppAAT1) did not correlate with ester and lactone production during postharvest peach ripening [37]. We can confirm this result as ppAAT1 is represented in our microarray (PPN018D08) and it poorly or moderately correlates with lactones and lineal esters (r between 0.27 and 0.60, Additional file 8: Table S5).

Correlation analyses revealed a CYP, PPN070H11, which is highly associated with the lactones in C2 (Table 1). CYPs represent the largest family of genes involved in plant metabolism, which include enzymes that are capable of epoxidizing or directly hydroxylating fatty acids. A CYP77A4 (belonging to the CYP77 subfamily) from *Arabidopsis* catalyzes the epoxidation of free oleic acid (18:1) to form 9,10 epoxyestearic acid [49], which has been proposed to be the precursor of γ-dodecalactone in peach [16]. Other CYPs (belonging to the CYP71 family), which are capable of epoxydating unsaturated C18 fatty acids (18:2 and 18:3) as phosphatidylcholine esters, have been characterized in a *Euphorbia* species [50]. Moreover, the position of the introduction of the hydroxyl group is crucial to producing a long hydroxy fatty acid, which, after β-oxidation, will produce the proper hydroxy acid molecule. The CYP703A2 (CYP703 subfamily) from *Arabidopsis* can catalyze the hydroxylation of saturated medium chain fatty acids (C10-C14) with a preference for hydroxylate at position C7 [51], and can therefore produce hydroxy fatty acids, which could be precursors for lactone generation after β-oxidation and cyclation. According to phylogenetic analyses (data not shown), the CYP identified here belongs to the CYP72A subfamily. CYP72A subfamily members are quite diverse, and most of them have unknown functions [52]. The CYP72 clan is also associated with the metabolism of other fairly hydrophobic compounds besides fatty acids, such as isoprenoids. Other members have been associated with the catabolism of hormones (brassinosteroids and gibberellins) or with the biosynthesis of cytokinins [52]. Thus, we cannot exclude the possibility that this candidate gene (CYP, PPN070H11) could be involved in lactone production by, for example, controlling a hormone metabolism instead of participating directly in lactone biosynthesis.

The HCA also identified a gene, PP1002E07, with a homology to fatty acid desaturase (FAD), as being highly associated with 2-Hexen-1-ol acetate (E)- (Additional file 9: Figure S4, Table 1). Nevertheless, this gene also correlated well with lactones (r= 0.65 to 0.89, Additional file 8: Table S5) and, in some cases, has correlation coefficients higher than those of 2-Hexen-1-ol, acetate (E)- (r=0.73, Additional file 8: Table S5). The *Arabidopsis* homolog of this gene is an ω-6 fatty acid desaturase, which catalyzes the reduction of oleic acid (18:1) esterified to the sn-2 position of the membrane lipid phosphatidylcholine (PC) to linoleic acid (18:2). Yet desaturase genes also share a good homology with other fatty acid modification enzymes, like hydroxylases and epoxidases [53]. For example, a hydroxylase that can hydroxylate oleic acid (18:1) and is linked to PC to form ricinoleic acid (18:1-OH) has been described in castor plants [54]. In addition, a bifunctional enzyme that can both catalyze hydroxylation and desaturate the same fatty acid has been reported in a *Brassicacae* species [55]. Moreover, it has been demonstrated that a plant desaturase can be converted into a hydroxylase, and *vice versa,* by swapping a few specific amino acids by targeted mutagenesis [56]. Since hydroxylase, desaturase, and epoxidase activity could lead to the production of hydroxy acids (Figure 6A), we expressed the functional protein encoded by PP1002E07 in yeast to clarify the reaction catalyzed by the putative encoded enzyme. Yeasts expressing the peach ORF gene PP1002E07 accumulated linoleic acid (18:2) when oleic acid (18:1) levels were reduced (Figure 5), indicating that the candidate gene has ω-6 Oleate desaturase activity. No ricinoleic acid was produced in the yeast expressing the candidate gene, suggesting that the encoded protein is not a bifunctional enzyme. Since no enzymes in plants have been shown to simultaneously desaturate and epoxidate fatty acid [53], our results suggest that the candidate gene identified herein is a monofunctional ω-6 Oleate desaturase.

Linoleic acid (18:2) may be further desaturated to linolenic acid (18:3), and both compounds could enter the Lipoxygenase (LOX) pathway to be catabolized into C6 and C9 alcohols, which are the substrates of AAT enzymes in the synthesis of a variety of esters (Figure 6A, [15]). Moreover, double bonds of unsaturated fatty acids could undergo the introduction of oxygen by hydratation to form hydroxy acids, which could be biosynthetic precursors of lactones (Figure 6A). In a previous report, we suggested that lipid-derived compounds and lactones are inversely regulated in peach fruit [11]. An analysis of the current data set also revealed lactones, specifically those in C2 and C3, showing strong inverse correlations with lipid-derived compounds belonging to C11 and C12 (Figure 4A). This is in agreement with previous results indicating that lactone content increases during maturity and ripening, while lipid-derived compounds decrease [35-38]. The fact that ω-6 Oleate desaturase positively correlates with lactones, i.e., its levels increase during ripening, while lipid-derived compounds decrease, seems to suggest that the formation of lipid-derived compounds downstream of linoleic acid (18:2) is regulated during peach ripening, and that this pathway can feed into the precursors for lactone synthesis.

Beside CYPs and desaturase-like epoxidases, a third kind of enzyme, Peroxygenase (PXG), which can catalyze the epoxygenation of fatty acids, has been reported in plants. PXGs share no structural similarity with either peroxidase or cytochrome P450, but instead do so with caleosin, a small oil body-associated protein with both heme- and calcium-binding motifs [57]. Here, a caleosin-related protein (PP1004C02), showing a strong correlation with g-jasmolactone, was indentified (Table 1, Additional file 10: Table S6). Recently, a PXG capable of epoxidating free oleic acid (18:1) was described in oat seeds [58]. It is interesting to note that PXG has potential kinase phosphorylation sites [59], and our approach also identified kinases that correlate well with C3 (PPN010B11, PPN069F09).

PXG, like FAD and CYPs, is found in plant microsomes, which implies that it could be associated with the endoplasmatic reticulum *in vivo*[50,59]*.* Interestingly, two SNARE genes associated with the endomembrane trafficking system (PP1004F06 and PPN031F12) also correlated with the C3 volatile cluster (Table 1, Additional file 10: Table S6), indicating the co-activation of both structural and metabolic enzymes.

It has been proposed that highly correlated metabolites in replicated experiments reflect the existence of a common regulatory mechanism in the system under study [60]. In this sense, our approach identifies several transcription factors relating to volatile clusters C1 (PP1009A02, PPN066C05) and C2 (PPN010C01), which are tentatively involved in the regulation of volatile production during peach fruit ripening.

The highly stringent criteria used in our approach indicate that our candidate genes are unlikely to be the result of random correlations. In any case, further functional studies are required to unequivocally prove the implication of the candidate gene in controlling the proposed volatile accumulation.

### Selection of candidate genes for other aroma-related compounds

Candidate genes for other minor aroma volatiles in peach have also been discovered. Ethyl acetate has been described to have a “fruity” aroma [61] and has also been proposed to be regulated independently of other esters in peach [11]. Here we demonstrate that the HCA identifies an enolase (phosphopyruvate hydratase, PPN071H07), which is highly related to the accumulation of both Ethanol and its acetate ester, Ethyl acetate (Additional file 9: Figure S4, Table 1). Enolase catalyzes one of the final glycolysis steps (EC. 4.2.1.11), which generates Pyr as an end product (Figure 6B). As previously mentioned, Pyr is also converted into acetaldehyde by the action of Pyr decarboxylase [48]. From acetaldehyde, the pathway splits into two branches, one producing ethanol and the other acetyl-CoA (Figure 6B, [48]). Ethyl acetate can be formed by the condensation of ethanol with Acetyl-CoA by a putative AAT, which is yet to be described. The correlation between ethanol and ethyl acetate with the enolase suggests that this gene is a good candidate for driving the production of glycolysis end products, i.e., Pyr, into aroma-contributing compounds.

The aroma of Benzeneacetaldehyde is described as “green” (Additional file 1: Figure S1) and could, therefore, confer immature fruit notes. Accordingly, the levels of this compound decreased during the ripening of the ‘Granada’ genotype (Figure 1) and after the shelf-life ripening in ‘MxR_01’ (Figure 2), which is in accordance with the shift of aroma from immature to ripe fruit. The correlation network analysis reveals that putative tyrosine (Tyr) aminotransferase (PPN018G03) correlates well (r=0.85) with this volatile, which is, in turn, related to other phenolic volatiles (Benzaldehyde and Benzyl chloride, Figure 1, Figure 4). Two pathways for the biosynthesis of aromatic volatiles have been described in different fruits (Figure 6C). In tomato, Phenylalanine (Phe) undergoes two sequential enzymatic steps: a decarboxylation step followed by a deamination step to form Benzeneacetaldehyde [62]. In contrast, in melon, Phe first loses the amine groups by transamination reactions to then undergo a decarboxylation reaction [63]. Aromatic acid transaminase (CmArAT1), which is involved in Benzeneacetaldehyde biosynthesis in melon, is capable of catalyzing the transamination of either Tyr or Phe [63]. The gene identified herein (PPN018G03) is phylogenetically related to CmArAT1 (data not shown), suggesting that it may have both transaminase activities. Therefore, we hypothesize that Benzeneacetaldehyde in peach is produced by a similar transaminase pathway to that in melon (Figure 6C).

As previously mentioned, the catabolism of linoleic and linolenic acid by the LOX/HPL pathway produces the so-called lipid-derived compounds [15]. As some of these volatiles are described to confer “green” aromas (Additional file 1: Figure S1), which is the typical aroma of freshly-cut grass, these volatiles have been traditionally associated with unripe fruits. Accordingly, we previously found that lipid-derived volatiles correlated highly among themselves and also with parameters that measure the peach ripening stage, indicating that high levels are linked to unripe fruit [11]. In addition, some of these compounds have been described to have other aromas, which could even be unpleasant when present in fruit, these being “fermented”, “spicy”, “fatty”, or even “chemical” (Additional file 1: Figure S1). In the data set used herein, these compounds are grouped into two highly interrelated clusters (C11 and C12, Figure 1, Figure 4A). The correlation network analysis (Additional file 13: Figure S5) reveals that a transcription factor (PPN019F11) strongly correlates with the expression levels of a gene with no homolog in *Arabidopsis* (PPN023E05) which, in turn, is highly correlated to a lipid δ 9 desaturase (PPN052H12). This lipid desaturase strongly and inversely correlates with lipid-derived compounds Furan 2-pentyl- and Hexanal. It is possible that the lipid desaturase catalyzes the formation of unsaturated lipids, which are not substrates for the LOX/HPL pathway, thus driving the carbon allocation toward other products. If this were the case, this gene would be a candidate for reducing lipid-derived compound production. Four candidate genes for C12 volatiles were selected because they correlated with 2,4-Heptadienal (E,E)- in the HCA (Additional file 9: Figure S4). Fatty acids are stored as triacylglycerides, so acyl hydrolases should free them to enter the LOX/HPL pathway [15]. One candidate gene is an esterase homolog (PPN033D11) that may be involved in the supply of fatty acids to the LOX/HPL pathway that produces lipid-derived volatiles, which makes it an interesting target for decreasing undesired aromas in peach fruit.

Cluster C4 is formed by two volatiles deriving from the terpenoid biosynthesis pathway and by four volatiles of unknown origin (Figure 1, Additional file 1: Figure S1). Our approach was able to identify the genes associated with C4 volatiles, although their functional annotations do not suggest a mechanism (Table 1, Additional file 10: Table S6). Nonetheless, it is quite likely that hormones are involved in the regulation of these compounds since gibberellin- and auxin-responsive proteins were identified (PP1000F05 and PP1009D02, respectively), as was aminopeptidase P (PPN017B08; Table 1, Additional file 10: Table S6). In *Arabidopsis*, it has been demonstrated that auxin signaling can be blocked by auxin-transport inhibitors, and that the specific Aminopeptidase P regulates hormone perception by degrading these inhibitors [64].

Our “omics” approach could be improved by including additional samples, i.e., fruits from other genotypes or those subjected to other treatments. For example, it has been demonstrated that low temperature storage and hot water treatments lower the volatile content of peach, while other quality parameters, e.g., flesh firmness, remain unaffected [46,65]. Thus, if the volatile reduction is due to changes in gene expression, these treatments may be used to unlink both processes and therefore improve gene identification by co-regulation patterns. Although further experiments are required to unequivocally prove some of the links between the genes and volatile compounds found herein, we provide empirical data, which may prove useful to boost gene-function discovery studies in peach fruit.

## Conclusions

In the present study, a hypothesis-free approach based on correlation analyses was applied to discover genes that are highly associated with the main aroma-contributing compounds of peach fruit. Consequently, a set of candidate genes for lactones, esters, terpenoids, phenolics and lipid-derived volatiles was identified. Some have functional annotations, which clearly suggest a possible role in controlling the production of related volatiles, which qualifies them as strong candidates to be selected for functional studies. In addition, for one of the candidate genes, we demonstrate that the encoded protein possesses enzymatic activity that produces a volatile peach precursor. Although our main interest was related to the practical implications of our results, the data provided herein not only support a previous hypothesis related to the physiology and metabolism of peach fruit, but also suggest novel ones. Although more studies are required to unequivocally prove the associations found herein, we provide experimental evidence and data analyses that support the role of a number of candidate genes in the control of volatile compounds, some of which may prove useful as targets for peach improvement and/or biotechnology industry interests.

## Competing interests

The authors declare that they have no competing interests.

## Authors’ contributions

GS conceived and designed the work, performed the metabolomics and microarray experiments, analyzed the data, and wrote the manuscript. MV-C and JJS performed the yeast expression experiment and the fatty acid analysis. AM and MLB contributed reagent material and analysis tools. AG conceived, designed, and supervised the work. All authors read and approved the final manuscript.

## Supplementary Material

Additional file 1: Figure S1 Volatile compounds analyzed in this study. For each volatile, the cluster that they belongs to according to Figure 1 (upper left corner), the chemical structure, the CAS number, the group and the odor description are shown. n.a., not available. *Bold indicates those volatiles whose retention time was verified by an authentic standard. **References for odor descriptions are: 1, Derail et al., 1999 [34]; 2, Guillot et al., 2006 [61]; and w, http://www.thegoodscentscompany.com. Click here for file

Additional file 2: Table S1 Genes analyzed by qRT-PCR analysis. For each gene, the microarray id (id), the identifier of the ChillPeach database (unigene id), the functional annotation, and the most similar gene from the peach genome sequence (Match) are shown. The forward and reverse primers and amplicon length are indicated for each gene tested. Validation indicates the Pearson correlation coefficient (PCC) between the microarray and qRT-PCR data for each gene. The gene profile was considered validated if PCC was higher than 0.7. Norm: indicates the gene used as normalizator in the qRT-PCR analysis. The gene annotated as “0.00E+00” indicate that either no homologue was found or the homologue found has unknown function. For a detailed description of ChillPeach unigene functional annotation see Ogundiwin et al. [5]. Click here for file

Additional file 3: Figure S2 Maturity time-course series of the ‘Granada’ and ‘MxR_01’ genotypes. A) Color index B) Firmness C) Weight D) Soluble Solids Content (SSC) E) CO2 consumption F) Ethylene production. Bars represent the LSD range. Click here for file

Additional file 4: Figure S3 Comparison of volatile contents in ‘Granada ’and ‘MxR_01’ at commercial maturity stage (S4). The values are expressed as fold changes on the Log2 scale. The positive region of the y-axis was used for values higher in ‘Granada’ as compared to ‘MxR_01’, and the indicated fold change is ‘Granada’/‘MxR_01’, while the negative region is used for values that are higher in ‘MxR_01’ as compared to ‘Granada’, and the indicated fold change is ‘MxR_01’/ “Granada”. All the differences are significant (p<0.05). Click here for file

Additional files 5: Table S2 Comparison of volatile content in ‘Granada’ and ‘MxR_01’ after shelf-life simulation (S4+SL). The fold change between genotypes is shown. The ANOVA p value for each volatile compound is indicated. Click here for file

Additional file 6: Table S3 Genes showing the same trends after the shelf-life condition in both genotypes. The fold change (up-regulated: S4+SL/S4; down-regulated: S4/S4+SL) for the ‘Granada’ and ‘MxR_01’ genotypes is indicated. For each gene, the microarray id (id), the unigene id and annotation and the most similar gene predicted in the peach genome (Match) are shown. The most similar *Arabidopsis* gene with the GO molecular function, the description, and the E-value are also provided. Click here for file

Additional file 7: Table S4 Genes affected by the shelf-life response with a 2-fold cut-off. The fold change for up-regulated (S4+SL/S4) and down-regulated (S4/S4+SL) genes in both genotypes is shown. The number of genes for each list are indicated in parentheses. For each gene, the microarray id (id), the unigene id and annotation and the most similar gene predicted in the peach genome (Match) are shown. The most similar *Arabidopsis* gene with the GO molecular function, the description and the E-value are also provided. n.f.: not found. Click here for file

Additional file 8: Table S5 Correlation matrix of the complete dataset. The Pearson correlation coefficient is shown. Click here for file

Additional file 9: Figure S4 Hierarchical cluster analysis for identifying the genes correlating with the 52 VOCs. A) The heatmap and cluster analyses of the gene-volatile data set (4348 genes and 52 volatiles). Three replicates per stage are shown. Data are expressed as the log2 of a ratio (sample/common reference). B) Details of the HCA where volatile compounds are present with genes. Volatiles are indicated with a red letter. For each gene, the id and unigene annotation and identifier are provided (in parentheses) when available. Three replicates per stage are shown. Sub-clusters are named according to the volatile members that they have. For example, the volatiles of C1, according to Figure 1, appear in three sub-clusters named C1.1, C1.2, and C1.3. Click here for file

Additional file 10: Table S6 Extended description for the candidate genes identified. Besides the information provided in Table 1, in this table, the ChillPeach database identifier (Unigene ID) and the localization of the gene in the peach genome is shown. The most similar *Arabidopsis* gene with its description and E-value are also provided. n.f.: not found. The genes annotated as “0.00E+00” indicate that either no homologue was found or the homologue found has unknown function. For a detailed description of ChillPeach unigene functional annotation see Ogundiwin et al. [5]. Click here for file

Additional file 11: Table S7 The ten genes that best correlated with γ-decalactone (upper) and γ-jasmodecalactone (bottom). For each gene, the microarray id (id), the unigene id and annotation, the Pearson Correlation coefficient (PC), and the most similar gene predicted in the peach genome are shown (Match). The most similar *Arabidopsis* gene with the GO molecular function, the description, and the E-value are also provided. n.f.: not found. The gene annotated as “0.00E+00” indicate that either no homologue was found or the homologue found has unknown function. For a detailed description of ChillPeach unigene functional annotation see Ogundiwin et al. [5]. Click here for file

Additional file 12: Table S8 Sub-cluster of genes obtained by CNA. For each gene, the microarray id (id), the unigene id and annotation, and a set of topological network parameters of its node are shown. The gene annotated as “0.00E+00” indicate that either no homologue was found or the homologue found has unknown function. For a detailed description of ChillPeach unigene functional annotation see Ogundiwin et al. [5]. Click here for file

Additional file 13: Figure S5 Correlation network of VOCs with genes correlating with the compounds of clusters C4 to C13. A) Network of VOCs and genes. The nodes representing volatiles are colored according to the cluster that they belong to (according to Figure 1). Genes are represented as gray nodes. Edges are colored according to their strength. The edge codification is indicated to the right of the network. Node size indicates its connectivity. The bigger the node, the higher the connectivity measured with the node degree (i.e., the number of edges connecting the node). A sub-cluster of genes is indicated with E. **B)** Magnification of volatile groups (C4, C11, C12, C5, and C13) showing the interactions with genes in detail. The genes annotated as “0.00E+00” indicate that either no homologue was found or the homologue found has unknown function. For a detailed description of ChillPeach unigene functional annotation see Ogundiwin et al. [5]. Click here for file

Additional file 14: Figure S6 Profile of the candidate gene (PPN001H09) expression assayed by microarray (left) and qRT-PCR (right) analysis. For both analyses, the Relative Quantitation (RQ) in arbitrary units is shown. The y-axis is on the log10 scale. Click here for file

Additional file 15: Figure S7 Alignment of PpFAD 1B_6 with FAD-type enzymes. Conserved amino acids are shaded in red. Open boxes indicate the predicted transmembrane domains (TM). The three His motifs (one HXXXHH and two HXXHH) are underlined. Sequence alignment was performed with MegAlign (DNAStar). PpFAD 1B-6, *Prunus persica* Fatty Acid Desaturase allele 1B-6; AtFAD2, *Arabidopsis thaliana*, Fatty Acid Desaturase type 2; RcFAH, *Ricinus communis* Fatty Acid Hydroxylase; LfFAHD, *Lesquerella fendleri* Fatty Acid Hydroxylase/Desaturase; CpFAE, *Crepis palaestina* Fatty Acid Epoxygenase. For AtFAD2, RcFAH, LfFAHD, and CpFAE, the NCBI accession numbers are provided following each name. Click here for file

Additional file 16: Table S9 Composition of fatty acid in yeast expressing PpFAD_1B-6. The % of total fatty acid content is shown for palmitic acid (16:0), palmitoleic acid (16:1), estearic acid (18:0), and ricinoleic acid (18:1-OH). No significant differences in fatty acid levels between treatments were detected by ANOVA (p<0.05), indicated by the same letter. The inducing and non-inducing conditions are indicated by + and -, respectively. The mean of three determinations (n=3) are shown. n.d.: not detected. Click here for file
