# Supplementary material for: An integrative “omics” approach identifies new candidate genes to impact aroma volatiles in peach fruit
Source: BMC Genomics. 2013 May 23;14:343. doi: 10.1186/1471-2164-14-343 (PMC3685534; doi:10.1186/1471-2164-14-343)
Supplement: Additional file 1: Figure S1 — Volatile compounds analyzed in this study. For each volatile, the cluster that they belongs to according to Figure 1 (upper left corner), the chemical structure, the CAS number, the group and the odor description are shown. n.a., not available. *Bold indicates those volatiles whose retention time was verified by an authentic standard. **References for odor descriptions are: 1, Derail et al., 1999 [34]; 2, Guillot et al., 2006 [61]; and w, http://www.thegoodscentscompany.com. [file 1471-2164-14-343-S1.pptx]

## Slide 1
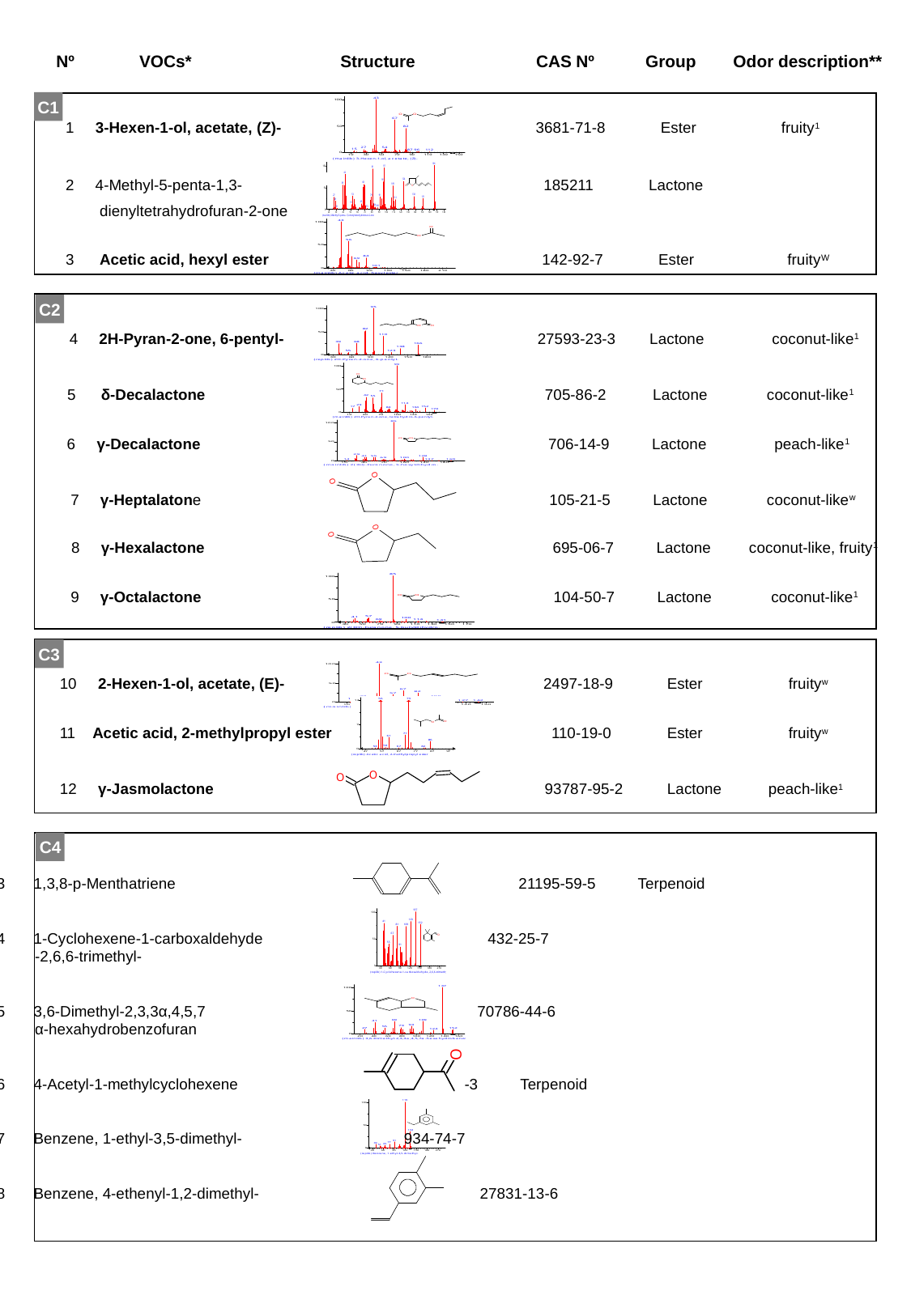

Nº VOCs* Structure CAS Nº Group Odor description**
C1
1 3-Hexen-1-ol, acetate, (Z)- 3681-71-8 Ester fruity1
2 4-Methyl-5-penta-1,3- 185211 Lactone
 dienyltetrahydrofuran-2-one
3 Acetic acid, hexyl ester 	 142-92-7 Ester fruityW
C2
4 2H-Pyran-2-one, 6-pentyl-	 27593-23-3 Lactone coconut-like1
5 δ-Decalactone 705-86-2 Lactone coconut-like1
6 γ-Decalactone 706-14-9 Lactone peach-like1
7 γ-Heptalatone 105-21-5 Lactone coconut-likew
8 γ-Hexalactone 695-06-7 Lactone coconut-like, fruity1
9 γ-Octalactone 104-50-7 Lactone coconut-like1
C3
10 2-Hexen-1-ol, acetate, (E)- 2497-18-9	Ester	fruityw
11 Acetic acid, 2-methylpropyl ester	 110-19-0	Ester	fruityw
12 γ-Jasmolactone	 93787-95-2 	Lactone peach-like1
C4
1,3,8-p-Menthatriene	 21195-59-5 Terpenoid
1-Cyclohexene-1-carboxaldehyde 432-25-7
 -2,6,6-trimethyl-
3,6-Dimethyl-2,3,3α,4,5,7 70786-44-6
 α-hexahydrobenzofuran
4-Acetyl-1-methylcyclohexene	 70286-20-3 Terpenoid
Benzene, 1-ethyl-3,5-dimethyl-	 934-74-7
18	Benzene, 4-ethenyl-1,2-dimethyl- 27831-13-6

## Slide 2
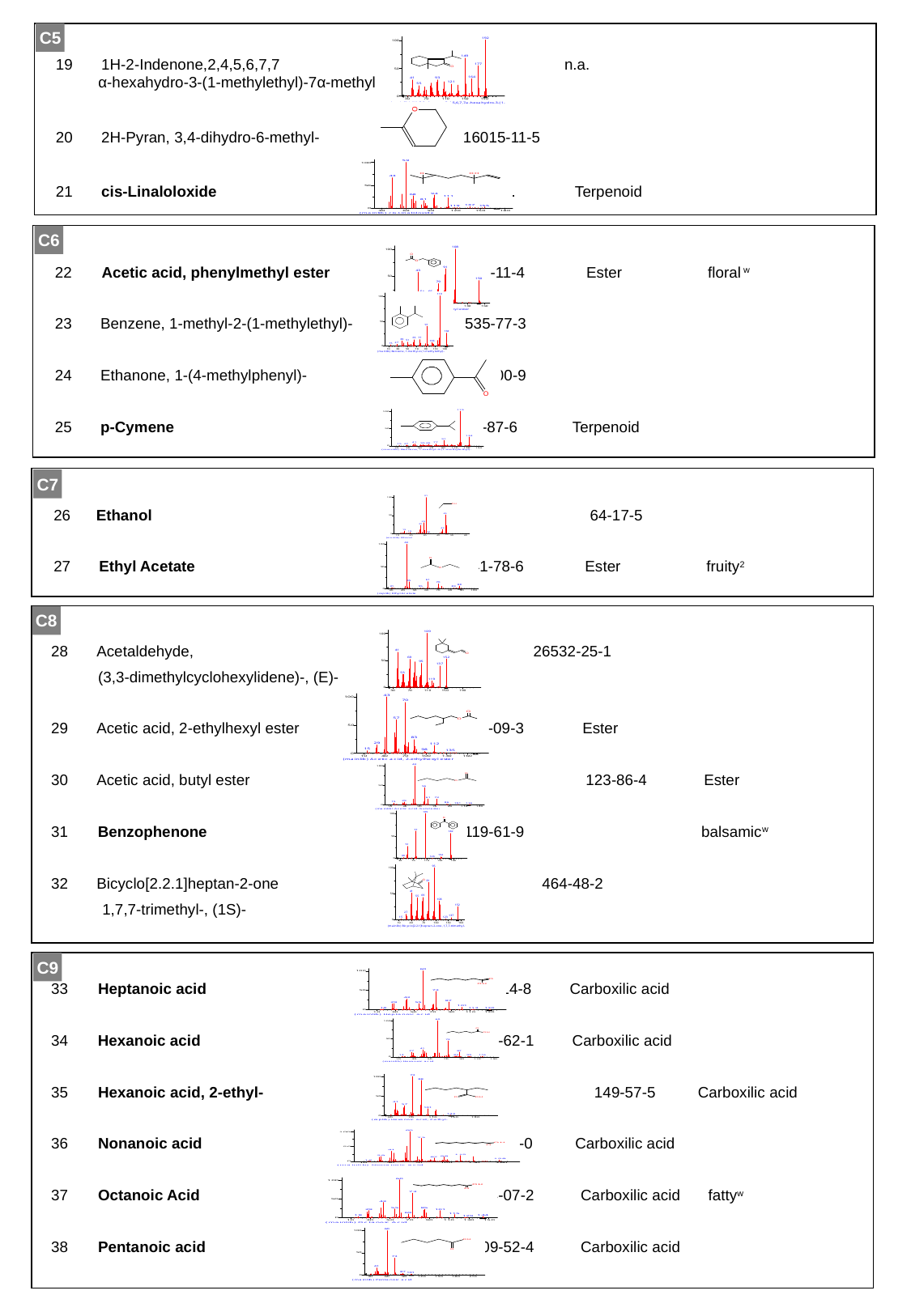

C5
1H-2-Indenone,2,4,5,6,7,7 n.a.
 α-hexahydro-3-(1-methylethyl)-7α-methyl
2H-Pyran, 3,4-dihydro-6-methyl-	 16015-11-5
21	cis-Linaloloxide	 n.a . Terpenoid
C6
22 Acetic acid, phenylmethyl ester 	140-11-4	Ester	floral w
Benzene, 1-methyl-2-(1-methylethyl)- 	535-77-3
Ethanone, 1-(4-methylphenyl)- 	122-00-9
25	p-Cymene 	99-87-6 Terpenoid
C7
26 Ethanol	 64-17-5
27	Ethyl Acetate	 141-78-6	Ester	fruity2
C8
Acetaldehyde, 26532-25-1
 (3,3-dimethylcyclohexylidene)-, (E)-
29	Acetic acid, 2-ethylhexyl ester	 103-09-3	Ester
Acetic acid, butyl ester	 123-86-4	Ester
31 Benzophenone	 119-61-9	 balsamicw
Bicyclo[2.2.1]heptan-2-one 464-48-2
 1,7,7-trimethyl-, (1S)-
C9
33 Heptanoic acid	 111-14-8 Carboxilic acid
34 Hexanoic acid	 142-62-1 Carboxilic acid
35 Hexanoic acid, 2-ethyl-	 149-57-5 Carboxilic acid
36 Nonanoic acid	 112-05-0 Carboxilic acid
37 Octanoic Acid	 124-07-2 Carboxilic acid	 fattyw
38 Pentanoic acid	 109-52-4 Carboxilic acid

## Slide 3
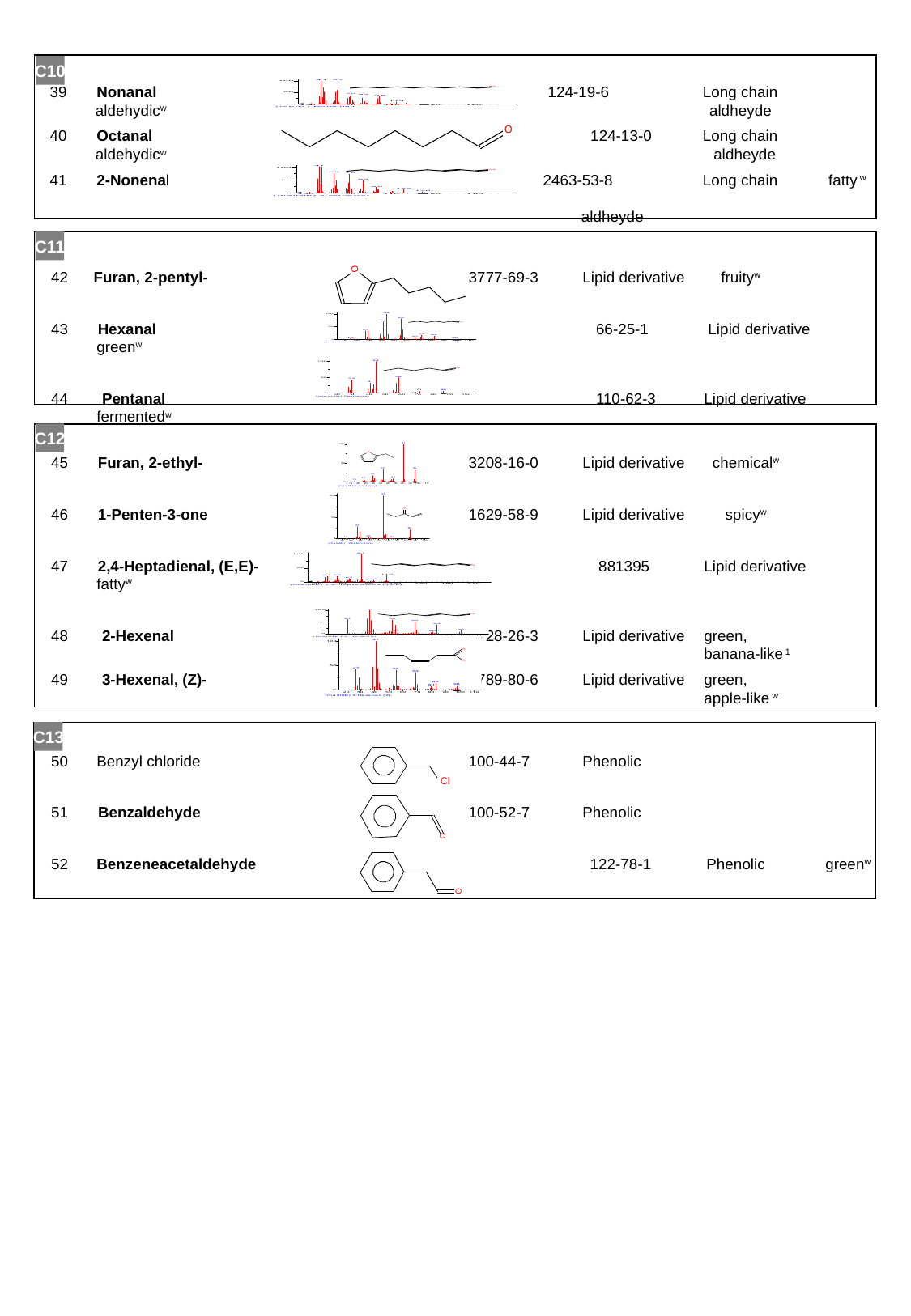

C10
39 Nonanal 124-19-6	Long chain aldehydicw 	 aldheyde
40 Octanal	 124-13-0	Long chain aldehydicw 	 aldheyde
41 2-Nonenal 2463-53-8	Long chain	 fatty w 					aldheyde
C11
42 Furan, 2-pentyl-	 3777-69-3	Lipid derivative 	 fruityw
43 Hexanal	 66-25-1 Lipid derivative	 greenw
44 Pentanal	 110-62-3	Lipid derivative	fermentedw
C12
45 Furan, 2-ethyl-	 3208-16-0	Lipid derivative	 chemicalw
46 1-Penten-3-one	 1629-58-9	Lipid derivative	 spicyw
47 2,4-Heptadienal, (E,E)-	 881395	Lipid derivative	 fattyw
48 2-Hexenal	 6728-26-3	Lipid derivative	green, 						banana-like 1
49 3-Hexenal, (Z)-	 6789-80-6	Lipid derivative	green, 						apple-like w
C13
Cl
Benzyl chloride	 100-44-7	Phenolic
51 Benzaldehyde	 100-52-7	Phenolic
52	Benzeneacetaldehyde	 122-78-1 Phenolic 	greenw
