# Supplementary figures and images for: An integrative “omics” approach identifies new candidate genes to impact aroma volatiles in peach fruit
Source: BMC Genomics. 2013 May 23;14:343. doi: 10.1186/1471-2164-14-343 (PMC3685534; doi:10.1186/1471-2164-14-343)

## Slide 1
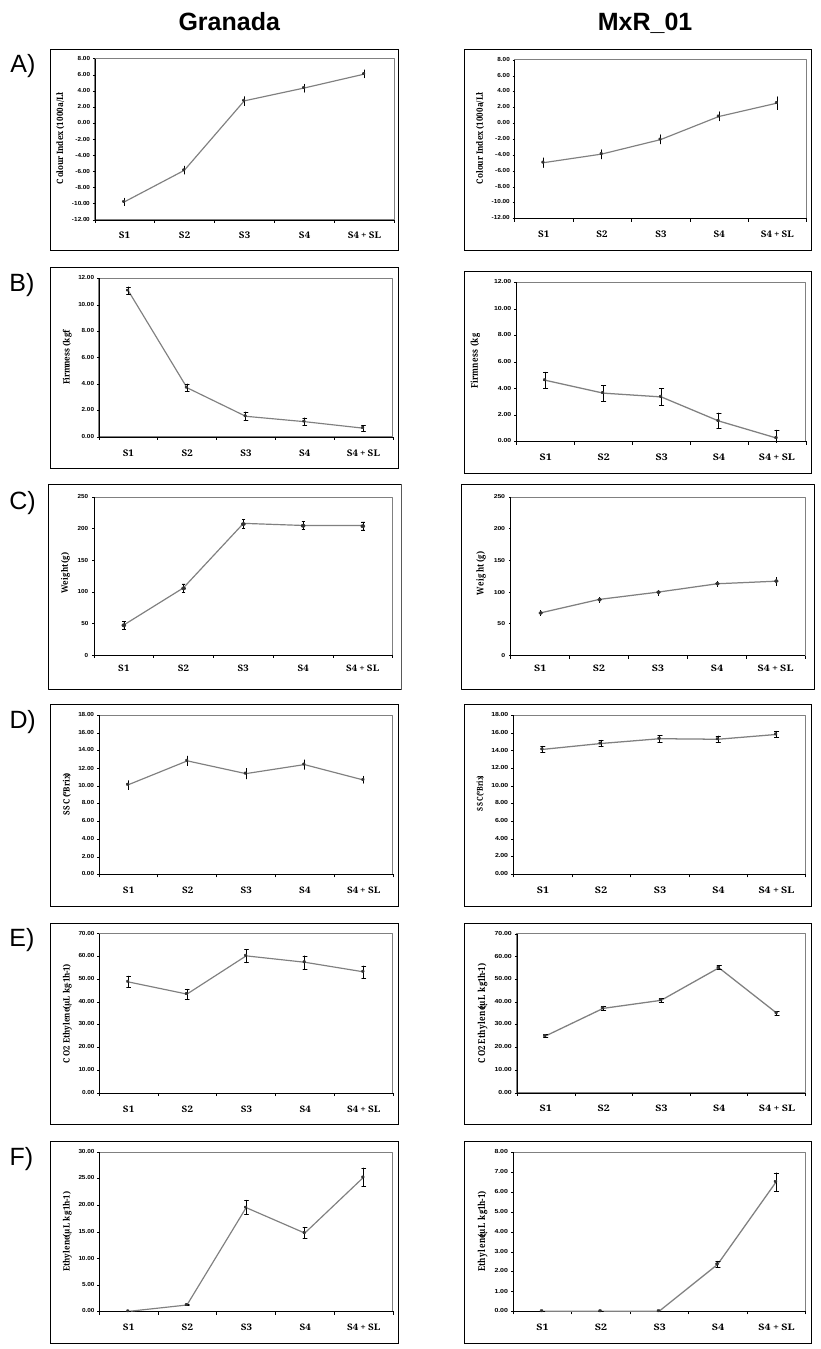

Granada
MxR_01
A)
B)
C)
D)
E)
F)

Supplement: Additional file 3: Figure S2 — Maturity time-course series of the ‘Granada’ and ‘MxR_01’ genotypes. A) Color index B) Firmness C) Weight D) Soluble Solids Content (SSC) E) CO2 consumption F) Ethylene production. Bars represent the LSD range. [file 1471-2164-14-343-S3.pptx]

## Slide 1
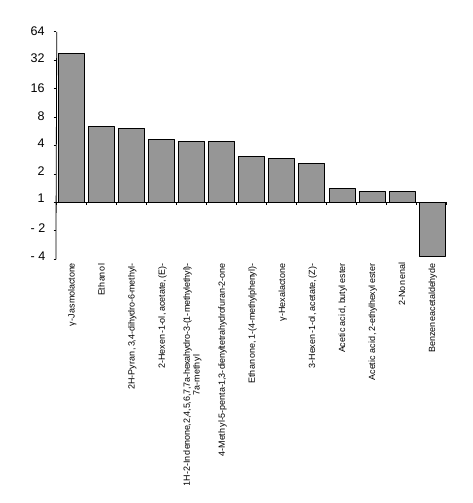

64
32
16
 8
 4
 2
 1
- 2
- 4

Supplement: Additional file 4: Figure S3 — Comparison of volatile contents in ‘Granada ’and ‘MxR_01’ at commercial maturity stage (S4). The values are expressed as fold changes on the Log2 scale. The positive region of the y-axis was used for values higher in ‘Granada’ as compared to ‘MxR_01’, and the indicated fold change is ‘Granada’/‘MxR_01’, while the negative region is used for values that are higher in ‘MxR_01’ as compared to ‘Granada’, and the indicated fold change is ‘MxR_01’/ “Granada”. All the differences are significant (p<0.05). [file 1471-2164-14-343-S4.pptx]

## Slide 1
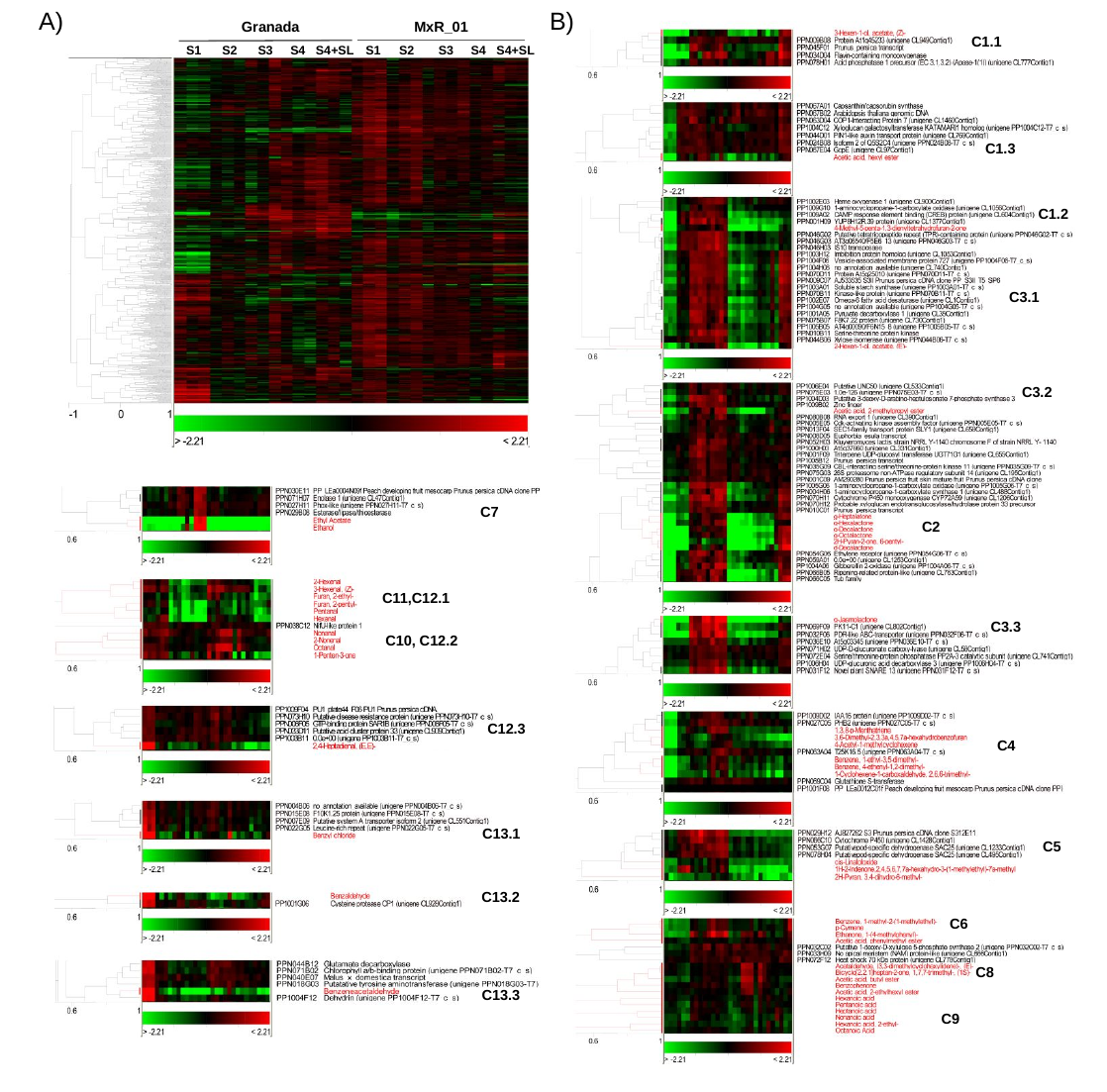

A)
B)
 Granada MxR_01
S1 S2 S3 S4 S4+SL S1 S2 S3 S4 S4+SL
C1.1
C1.3
C1.2
C3.1
C3.2
C7
C2
C11,C12.1
C3.3
C10, C12.2
C12.3
C4
C13.1
C5
C13.2
C6
C8
C13.3
C9

Supplement: Additional file 9: Figure S4 — Hierarchical cluster analysis for identifying the genes correlating with the 52 VOCs. A) The heatmap and cluster analyses of the gene-volatile data set (4348 genes and 52 volatiles). Three replicates per stage are shown. Data are expressed as the log2 of a ratio (sample/common reference). B) Details of the HCA where volatile compounds are present with genes. Volatiles are indicated with a red letter. For each gene, the id and unigene annotation and identifier are provided (in parentheses) when available. Three replicates per stage are shown. Sub-clusters are named according to the volatile members that they have. For example, the volatiles of C1, according to Figure 1, appear in three sub-clusters named C1.1, C1.2, and C1.3. [file 1471-2164-14-343-S9.pptx]

## Slide 1
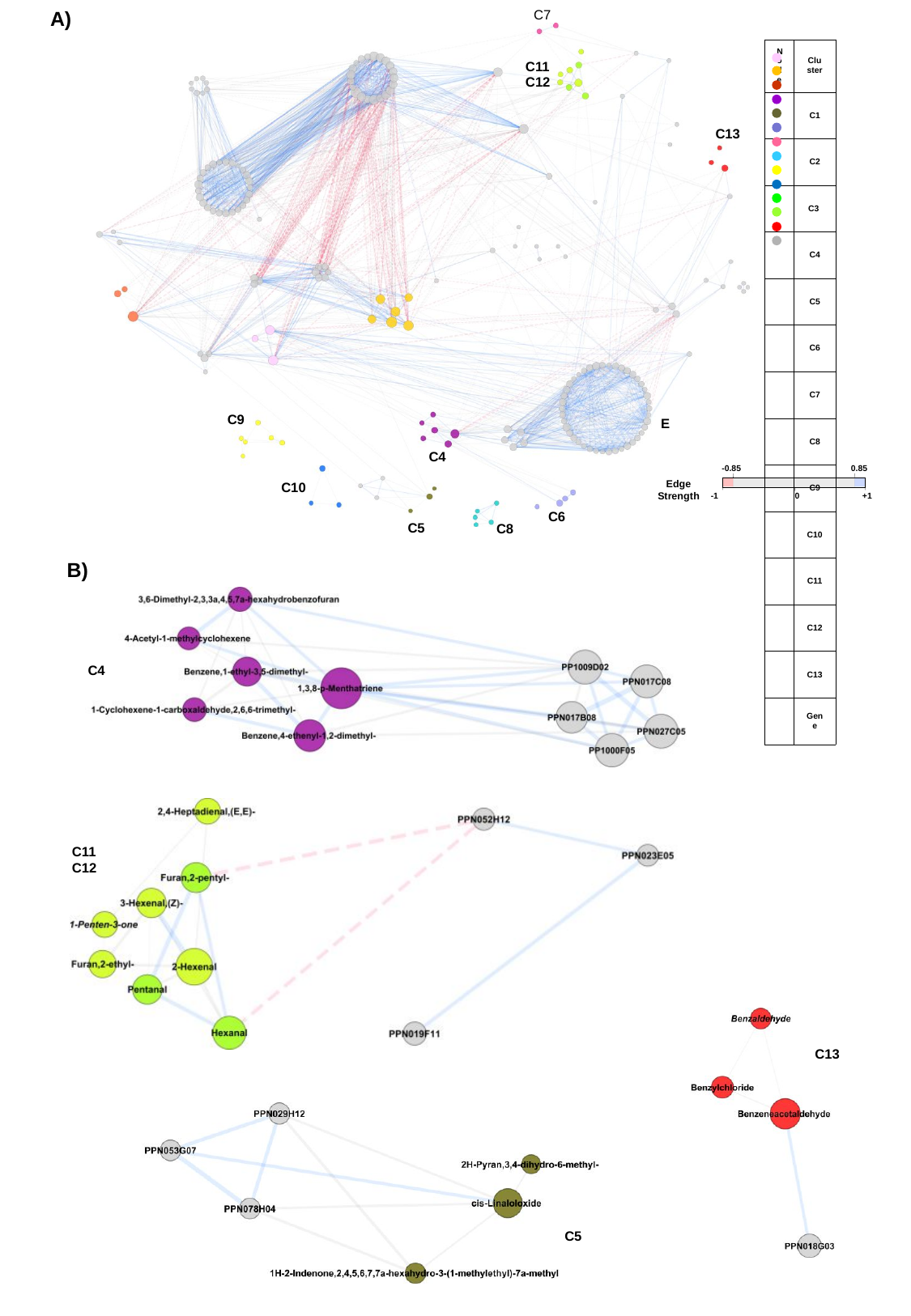

A)
C7
| Node | Cluster |
| --- | --- |
| | C1 |
| | C2 |
| | C3 |
| | C4 |
| | C5 |
| | C6 |
| | C7 |
| | C8 |
| | C9 |
| | C10 |
| | C11 |
| | C12 |
| | C13 |
| | Gene |
C11
C12
C13
C9
E
C4
 -0.85 0.85
Edge
Strength
-1 0 +1
C10
C6
C5
C8
B)
C4
C11
C12
C13
C5

Supplement: Additional file 13: Figure S5 — Correlation network of VOCs with genes correlating with the compounds of clusters C4 to C13. A) Network of VOCs and genes. The nodes representing volatiles are colored according to the cluster that they belong to (according to Figure 1). Genes are represented as gray nodes. Edges are colored according to their strength. The edge codification is indicated to the right of the network. Node size indicates its connectivity. The bigger the node, the higher the connectivity measured with the node degree (i.e., the number of edges connecting the node). A sub-cluster of genes is indicated with E. B) Magnification of volatile groups (C4, C11, C12, C5, and C13) showing the interactions with genes in detail. The genes annotated as “0.00E+00” indicate that either no homologue was found or the homologue found has unknown function. For a detailed description of ChillPeach unigene functional annotation see Ogundiwin et al. [5]. [file 1471-2164-14-343-S13.ppt]

## Slide 1
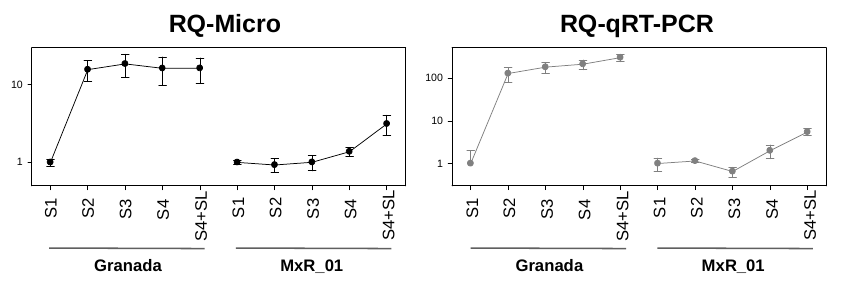

RQ-Micro RQ-qRT-PCR
 S1
 S2
 S3
 S4
 S4+SL
 S1
 S2
 S3
 S4
 S4+SL
Granada MxR_01
 S1
 S2
 S3
 S4
 S4+SL
 S1
 S2
 S3
 S4
 S4+SL
Granada MxR_01

Supplement: Additional file 14: Figure S6 — Profile of the candidate gene (PPN001H09) expression assayed by microarray (left) and qRT-PCR (right) analysis. For both analyses, the Relative Quantitation (RQ) in arbitrary units is shown. The y-axis is on the log10 scale. [file 1471-2164-14-343-S14.pptx]

## Slide 1
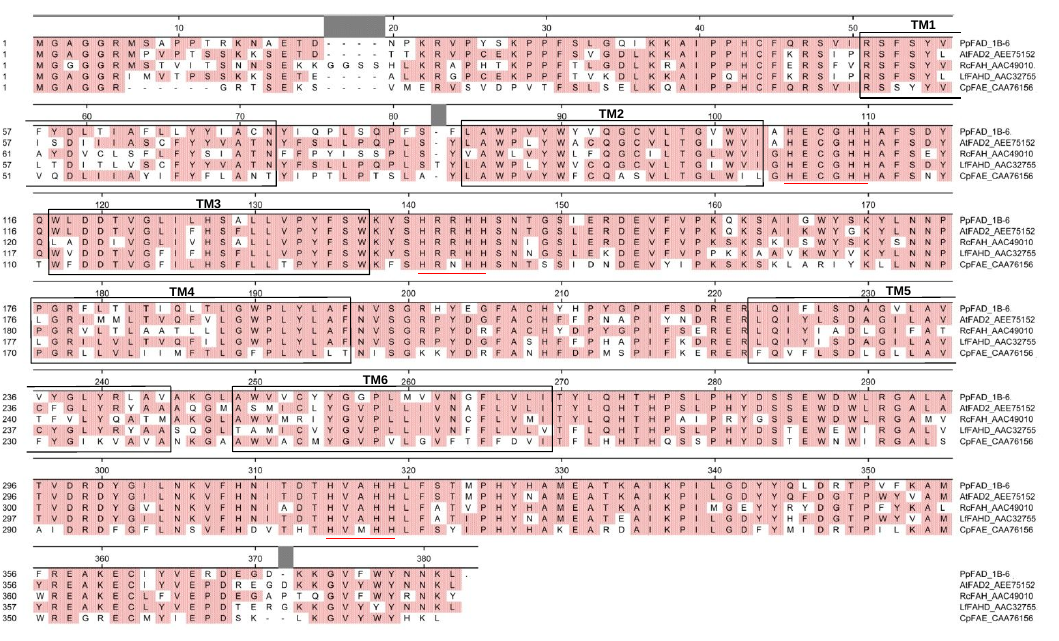

TM1
TM2
TM3
TM5
TM4
TM6

Supplement: Additional file 15: Figure S7 — Alignment of PpFAD 1B_6 with FAD-type enzymes. Conserved amino acids are shaded in red. Open boxes indicate the predicted transmembrane domains (TM). The three His motifs (one HXXXHH and two HXXHH) are underlined. Sequence alignment was performed with MegAlign (DNAStar). PpFAD 1B-6, Prunus persica Fatty Acid Desaturase allele 1B-6; AtFAD2, Arabidopsis thaliana, Fatty Acid Desaturase type 2; RcFAH, Ricinus communis Fatty Acid Hydroxylase; LfFAHD, Lesquerella fendleri Fatty Acid Hydroxylase/Desaturase; CpFAE, Crepis palaestina Fatty Acid Epoxygenase. For AtFAD2, RcFAH, LfFAHD, and CpFAE, the NCBI accession numbers are provided following each name. [file 1471-2164-14-343-S15.pptx]
